# Individual and joint effect of socioeconomic status and lifestyle factors on cancer in Korea

**DOI:** 10.1002/cam4.6359

**Published:** 2023-07-25

**Authors:** Chi Lan Tran, Kui Son Choi, Sun‐Young Kim, Jin‐Kyoung Oh

**Affiliations:** ^1^ Graduate School of Cancer Science and Policy National Cancer Center Goyang South Korea; ^2^ National Cancer Control Institute National Cancer Center Goyang South Korea; ^3^ Division of Cancer Prevention National Cancer Center Goyang South Korea

**Keywords:** cancer, joint effect, lifestyle factors, socioeconomic status

## Abstract

**Background:**

There is limited evidence on the individual and joint effect of socioeconomic status (SES) and unhealthy lifestyle on cancer. Therefore, this study aimed to examine the effects of these factors on cancer incidence and mortality.

**Methods:**

In this population‐based cohort study, income was used as the proxy of SES. A combined unhealthy lifestyle score was obtained using data on smoking, alcohol consumption, physical activity, and body mass index. Hazard ratios were estimated using a Cox proportional hazards model.

**Results:**

The study included data on 8,353,169 participants (median follow‐up period, 17 years). Although the association between low income and cancer incidence varied depending on cancer type, low income consistently increased the risk of cancer‐related death with a social gradient. Unhealthy behaviors increased the risk of cancer incidence and mortality, except for thyroid and breast cancer in women and prostate cancer in men. Compared with the wealthiest and healthiest individuals, the poorest and unhealthiest men and women showed 2.1‐fold (2.05–2.14) and 1.36‐fold (1.31–1.41) higher risk of cancer‐related death, respectively. The joint effect was most robust for lung, liver, head, and neck cancers in men and liver and cervical cancers in women; further, the effect was stronger with cancer‐specific mortality than with incidence.

**Conclusion:**

In conclusion, income and combined healthy lifestyle behaviors have individual and joint effects on cancer incidence and mortality. The effect varies by cancer type and sex.

## Graphical abstract

Using large population‐based data, this study shows the individual and joint effect of SES and lifestyle factors on cancer incidence and mortality, varied by cancer type and sex. Individuals of the lowest income and unhealthiest lifestyle experience the highest risk for cancer incidence and mortality. The effect appeared to be stronger with cancer‐specific mortality than with cancer incidence among both sexes, with men showing a more substantial effect than women.

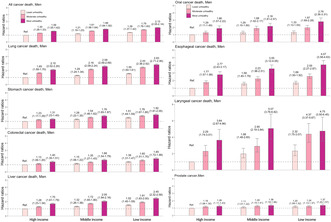

## 1 INTRODUCTION

Cancer is considered a disease of disparities. Several cancers occur more frequently among the poor, particularly cancers associated with tobacco use, chronic infections, and exposure to some dietary, reproductive, occupational, and environmental factors.^1^ Previous analyses among the United States and Western populations suggested that cancers of the lung,^2^, ^3^, ^4^, ^5^ upper aerodigestive tract,^2^, ^5^, ^6^, ^7^, ^8^ colorectal,^3^, ^4^ stomach,^3^, ^4^, ^8^ liver,^3^ and cervical cancer^3^, ^4^, ^6^ appear to be more frequent among lower SES groups. In contrast, other cancers happen more in the higher SES groups, including cancer of the breast,^4^, ^5^, ^6^ thyroid, prostate,^4^, ^6^ and melanoma.^4^, ^5^, ^6^ However, this higher incidence could result from a significant proportion of early‐stage cancer detected through cancer screening programs, especially for thyroid and prostate cancer, in which overdiagnosis and overtreatment have been a great concern.^9^, ^10^ In the United States, a study in 2016 revealed that early‐stage thyroid cancer, melanoma, prostate cancer, and female breast cancer were significantly higher among the affluent.^11^ On the other hand, late‐stage thyroid cancer and melanoma were not different among SES groups, and late‐stage prostate cancer and female breast cancer were significantly elevated among the poor.^11^ This may be caused by a high proportion of early‐stage cancer detected through cancer screening programs.

Socioeconomic gaps between people with cancer can be caused by disparities in lifestyle risk factors; the prevalence of smoking, high‐risk alcohol consumption, and physical inactivity is higher in lower SES groups.^12^ Smoking has been proven to have a strong causal relationship with cancers of the lung, oral cavity, larynx, esophagus, stomach, pancreas, colorectum, liver, kidney, ureter, bladder, cervix, ovary, and acute myeloid leukemia.^13^, ^14^ There are also strong dose–response relationships between alcohol consumption and cancers of the oral cavity, pharynx, larynx, esophagus, colorectum, liver, female breast, and intrahepatic bile duct.^13^ WCRF/IARC recommended that it is best not to drink for cancer prevention.^15^ According to the American Cancer Society, people who choose to drink, would limit consuming no more than two drinks per day for men, and one drink per day for women.^16^ There is also strong evidence that being physically active reduces the risk of all‐cause mortality and cancer of the bladder, breast, colon, endometrial, esophageal adenocarcinoma, renal, and gastric.^13^, ^17^, ^18^ Besides, a growing number of studies suggested that prolonged sedentary behavior increases cancer risk overall, with the most robust evidence for breast, colon, and endometrium cancers.^13^ Furthermore, postmenopausal breast, colorectum, endometrial, kidney, liver, esophageal, and pancreas cancers are strongly linked to overweight and obesity; while gall bladder, gastric, mouth, pharynx, larynx, ovary, and prostate cancers have a possible association.^13^ Therefore, the WCRF/IARC recommends maintaining a healthy weight (BMI 18.5–24.9) and having a healthy diet for cancer prevention.^15^

While single lifestyle factors are associated with adverse health outcomes, many studies provide evidence for how combinations of lifestyle factors have stronger associations with mortality and NCDs.^19^ Previous studies in Western countries use a healthy lifestyle score (HLIs‐assigning 0–4 points for each risk factor, then summing all points to create the combined score) incorporated multiple lifestyle risk factors, suggesting that the combination of a healthier lifestyle is associated with a lower risk of mortality and cancer.^20^, ^21^, ^22^, ^23^ Several other studies use a more straightforward method: assigning 1 point for each unhealthy or healthy lifestyle risk factor, then summing all points to create overall unhealthy or healthy scores, which also show similar results.^24^, ^25^, ^26^, ^27^

Moreover, a recent United Kingdom study indicated the joint effect of a combination of lifestyle behaviors and low income on cardiovascular disease (CVD) and all‐cause death, with a dose–response increased risk in most lifestyle strata and social groups when comparing to the combined reference group of the healthiest and wealthiest.^28^ Other studies in the United Kingdom and United States also showed that lower SES and less healthy lifestyles led to 2.65‐ to 3.53‐fold higher mortality risks than did higher SES and healthier lifestyles.^29^

However, these are based on mostly Western populations and limited health endpoints^28^, ^29^; results could differ in other populations and health outcomes due to different disease patterns, risk factors, and health policies. In South Korea (hereafter Korea), few studies have evaluated the effect of SES on cancer, and they were limited by their cross‐sectional design or lack of information on risk factors.^30^, ^31^, ^32^, ^33^ Moreover, the combined effect of multiple unhealthy behaviors on cancer and its joint effect with SES has not been sufficiently described.^34^ Therefore, using large, representative data, we evaluated the effect of SES, the effect of a combination of unhealthy lifestyle behaviors, and the joint effect of SES and unhealthy lifestyle on cancer incidence and mortality in South Korea.

## 2 MATERIALS AND METHODS

### 2.1 Data source

The data were based on a nationwide population‐based cohort provided by the Korea National Health Insurance Service (NHIS), a mandatory single‐insurer system covering the entire population of Korea.^35^ NHIS provides benefits to all beneficiaries (97%) and medical aid (3%) for healthcare services, including biennial health screening. Information on sociodemographic factors, self‐reported health behaviors, laboratory results, and healthcare utilization data from insurance claims were included.^35^, ^36^

### 2.2 Study population

**FIGURE 1:**
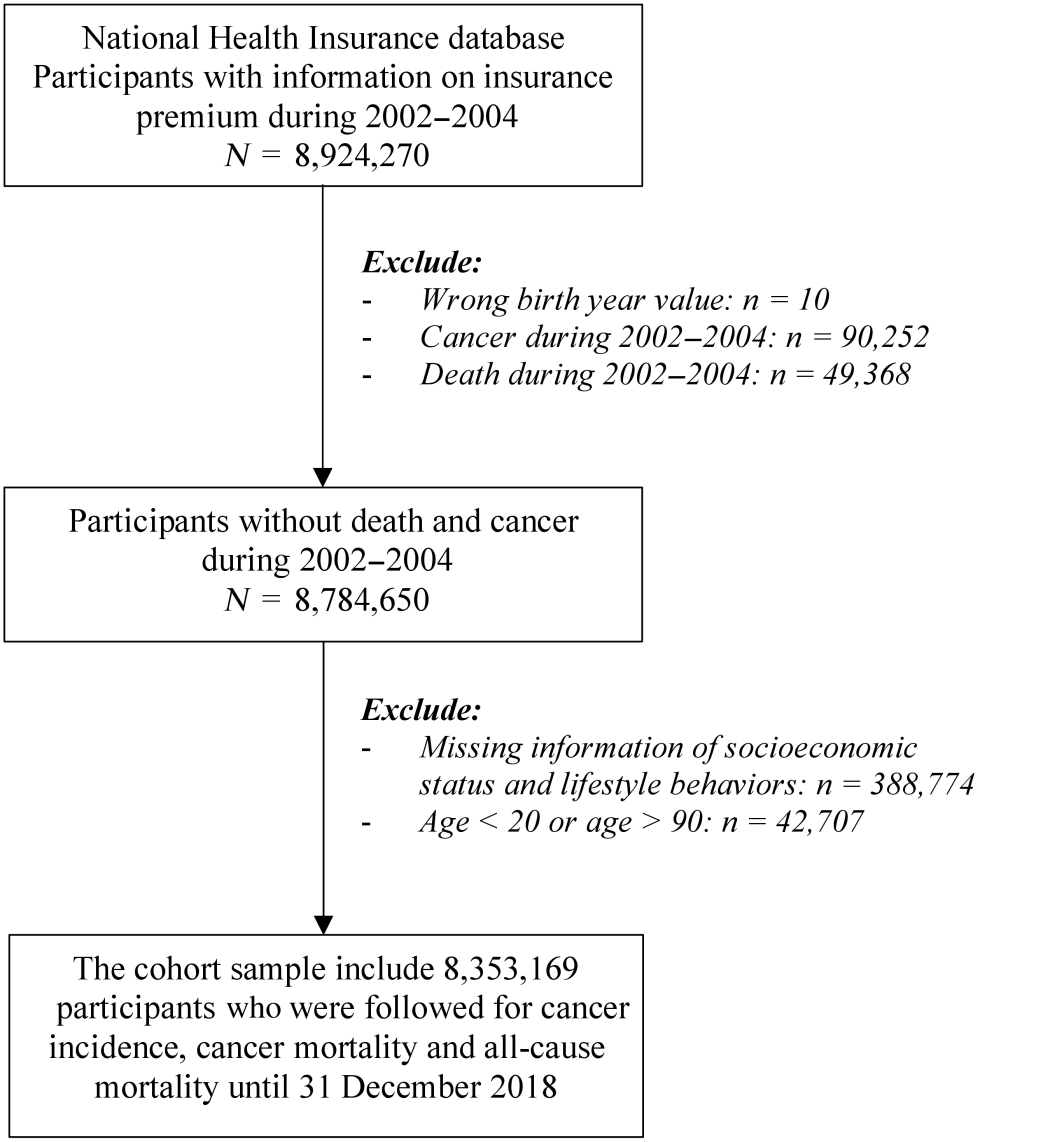
Flowchart of study.

We included 8,924,270 participants whose insurance premium information from 2002 to 2004 (baseline period) was available. We excluded those with incorrect birth‐years (*n* = 10). We created a cancer‐free cohort by excluding individuals diagnosed with cancer (*n* = 90,252) and those who died (*n* = 49,368) from 2002 to 2004. Individuals with missing baseline data on socioeconomic characteristics and lifestyle behaviors (*n* = 388,774) and those aged <20 or > 90 years (*n* = 42,707) were excluded. Finally, our analysis included 8,353,169 participants (4,974,821 men and 3,378,348 women, Figure S1). Figure 1 describes the flowchart of study.

### 2.3 Health outcomes

The outcomes of interest included cancer‐specific mortality and cancer incidence. All‐cause mortality was considered a secondary outcome. Cancer‐specific death was determined using the cause of death (ICD‐10: C00‐C99) derived from Statistics Korea. Cancer incidence was based on the ICD‐10 codes in the primary diagnosis and the NHIS registration code V193, a special code representing confirmed cancer diagnosis in NHIS database.^37^

For men, we focused on six major cancers: lung (C33 and C34), stomach (C16), colorectal (C18, C19, and C20), prostate (C61), liver (C22), and thyroid (C73) cancers.^38^ We were also interested in head and neck cancers (oral cavity [C00‐C14], esophageal [C15], and laryngeal cancers [C32]) which had previous evidence on social inequalities and associations with combined lifestyle risk factors.^7^, ^39^ For women, we focused on major cancers: breast (C50), thyroid (C73), colorectal (C18, C19, and C20), stomach (C16), lung (C33, C34), liver (C22), and cervical (C53) cancers.^38^

### 2.4 Socioeconomic status

Income was used as a proxy for SES and was identified using NHIS premiums.^40^ NHIS premiums are based on household income and are divided into 20 quintiles, with the higher quintiles corresponding to the more affluent participants. We divided the study population into five income classes from lowest to highest: Class 5 (1st–4th) Class 4 (5th–8th), Class 3 (9th–12th), Class 2 (13th–16th), and Class 1 (17th–20th).

#### 2.4.1 Lifestyle behaviors

Lifestyle behaviors including smoking status, alcohol consumption, and physical activity, were evaluated using structured questionnaires at general health screening during the baseline period. For smoking status, participants were asked whether they are current smokers (using question with three choices: (1) do not smoke; (2) former; (3) current). For alcohol consumption, the frequency (using question with five choices: (1) rarely drink; (2) drink 2–3 times a month; (3) drink 1–2 times a week; (4) drink 3–4 times a week; (5) drink almost daily) and amount of alcohol consumed were collected. Then we calculated the amount of alcohol consumed per day, which was then classified into three groups: none, light drinking, and heavy drinking (≥2 standard drinks/day for men and ≥1 standard drink/day for women; one standard drink = 14 g of alcohol).^16^ For physical activity, exercise frequency per week was recorded (using question with five answer choices: (1) do not, (2) 1–2 times, (3) 3–4 times, (4) 5–6 times, (5) almost daily). People who answered “do not” were considered physically inactive. Body mass index (BMI) was calculated directly using body weight and height measured at health institutions. Participants were categorized into underweight (body mass index [BMI] < 18.5), normal (18.5 ≤ BMI < 25), obese I (25 ≤ BMI < 30), and obese II (BMI≥30) based on the World Health Organization BMI standards for Asian populations.^41^ The combined unhealthy lifestyle score, which ranged from 0 (least unhealthy) to 4 (most unhealthy), was determined by assigning 1 point for the following: former or current smoking, heavy drinking, physical inactivity, and abnormal BMI (BMI < 18.5 or ≥ 25).

### 2.5 Other covariates

Other covariates included age, sex, area of residence, and insurance subscriber type using the first recorded information. Residential areas were divided into metropolitan cities (Seoul, Busan, Daegu, Incheon, Gwangju, Daejeon, and Ulsan) and provinces (other areas). Insurance subscriber types were classified into employment subscribers, local subscribers, and medical aid. The Charlson comorbidity index (CCI) was determined using data obtained from 2002 to 2003 to reflect participants' comorbidities. The ICD‐10 code B18 was used to identify participants with chronic viral hepatitis.

### 2.6 Statistical analysis

Descriptive analyses were performed to examine the characteristics and income groups of the study population. We used the Cox proportional hazards model to calculate adjusted hazard ratios (aHRs) and 95% confidence intervals (CIs) for the effect of income and combined unhealthy lifestyle behaviors on health outcomes. Follow‐up ranged from January 1, 2002 to diagnosis (for cancer incidence), death, or end of follow‐up (December 31, 2018), whichever came first. All analyses were conducted separately for men and women.

To examine the effects of income on health outcomes, the highest income group was set as the reference group. The model was adjusted for age, smoking status, alcohol consumption, physical activity, BMI, CCI, area, and insurance subscriber type as well as chronic viral hepatitis for liver cancer. The p for the linear trend was calculated by modeling the income variable as a continuous variable.

For the combined unhealthy lifestyle behaviors, the combined score was treated as a categorical variable, with five groups among men (0, 1, 2, 3, and 4) and four groups among women (0, 1, 2, and ≥3) due to the small number of women with four unhealthy behaviors. The healthiest group (score 0) was used as the reference group. The model was adjusted for age, CCI, income, area, insurance subscriber type, and chronic viral hepatitis for liver cancer. The p for the linear trend was calculated by modeling the combined score as a continuous variable.

For the joint effect of income and unhealthy behaviors on health, we classified participants into nine groups by income (low, middle, and high) and unhealthiness level (least unhealthy/moderate unhealthy/most unhealthy), as in previous studies.^28^, ^29^ Income was reclassified into low income (0th–8th), middle income (9th–14th), and high income (15th–20th) with relatively similar sizes to increase the statistical power. The combined unhealthy lifestyle score was also regrouped into three categories separately for men (0–1 as least unhealthy/2 as moderately unhealthy/3–4 as most unhealthy) and women (0 as least unhealthy/1 as moderately unhealthy/≥2 as most unhealthy). The healthiest and wealthiest individuals were used as reference. The model was adjusted for age, area, insurance subscriber type, CCI, and chronic viral hepatitis for liver cancer.

We also used logistic regression to calculate odds ratios (ORs) and 95% CIs of having individual and combined lifestyle behaviors among lower income groups compared with the highest income group. The model was adjusted for age, sex, area, insurance subscriber type, and CCI. The p for the linear trend was calculated by modeling the income variable as a continuous variable.

Statistical analyses were conducted using SAS Enterprise Guide Software (version 7.1, SAS Institute).

## 3 RESULTS

### 3.1 Characteristics

A total of 8,353,169 participants were included, with 4,974,821 men (59.56%) and 3,378,348 women (40.44%). The mean age was 43.75 years, with most participants aged 20–64. Men were more likely to be smokers, heavy drinkers, and have an abnormal BMI, while women were more likely to be physically inactive and underweight; they also had more low‐income participants and less employment subscribers (Table 1). Characteristics of each income group among men and women are described in Tables S1 and S2, and health outcomes among participants are described in Table S3.

**TABLE 1:** Characteristics of study participants.

Characteristics	Total		Men		Women	
	8,353,169		4,974,821 (59.56%)		3,378,348 (40.44%)	
	*N*	%	*N*	%	*N*	%
Age						
Mean (SD)	43.75 (13.54)		42.67 (12.63)		45.35 (14.64)	
20–39	3,405,575	40.77	2,274,828	45.73	1,130,747	33.47
40–64	4,242,822	50.79	2,371,747	47.67	1,871,075	55.38
65+	704,772	8.44	328,246	6.60	376,526	11.15
Smoking status						
Never	4,727,473	56.59	1,519,799	30.55	3,207,674	94.95
Former	831,113	9.95	770,870	15.50	60,243	1.78
Current	2,794,583	33.46	2,684,152	53.95	110,431	3.27
Alcohol consumption						
None	3,603,081	43.13	1,222,217	24.57	2,380,864	70.47
Light drink	3,841,287	45.99	2,942,805	59.15	898,482	26.60
Heavy drink^a^	908,801	10.88	809,799	16.28	99,002	2.93
BMI						
Mean (SD)	23.84 (3.19)		24.22 (3.04)		23.28 (3.33)	
<18.5	274,817	3.29	96,618	1.94	178,199	5.27
18.5–24.9	5,238,800	62.72	2,975,884	59.82	2,262,916	66.98
25.0–29.9	2,571,956	30.79	1,741,752	35.01	830,204	24.57
> = 30	267,596	3.20	160,567	3.23	107,029	3.18
Physical activity						
Do not exercise	3,915,398	46.87	1,863,282	37.45	2,052,116	60.74
Chronic hepatitis virus	355,874	4.26	232,329	4.67	123,545	3.66
Income (five groups)						
Class 5 (1th–4th)	1,230,687	14.73	542,452	10.9	688,235	20.37
Class 4 (5th–8th)	1,407,134	16.85	755,442	15.19	651,692	19.29
Class 3 (9th–12th)	1,957,897	23.44	1,243,251	24.99	714,646	21.15
Class 2 (13th–16th)	1,757,695	21.04	1,148,597	23.09	609,098	18.03
Class 1 (17th–20th)	1,999,756	23.94	1,285,079	25.83	714,677	21.16
Income (three groups)						
Low (1th–8th)	2,637,821	31.58	1,297,894	26.09	1,339,927	39.66
Middle (9th–14th)	2,770,251	33.16	1,778,180	35.74	992,071	29.37
High (15th–20th)	2,945,097	35.26	1,898,747	38.17	1,046,350	30.97
Insurance subscriber types						
Employment subscriber	6,005,553	71.90	3,850,104	77.39	2,155,449	63.80
Local subscriber	2,333,718	27.94	1,119,397	22.51	1,214,321	35.95
Medical benefit	13,898	0.16	5320	0.10	8578	0.25
Area						
Metropolitan city	4,020,628	48.13	2,415,312	48.55	1,605,316	47.52
Province	4,332,541	51.87	2,559,509	51.45	1,773,032	52.48
Unhealthy lifestyle scores						
0	1,321,676	15.82	488,077	9.81	833,599	24.67
1	3,488,303	41.76	1,770,875	35.60	1,717,428	50.84
2	2,654,593	31.78	1,888,131	37.95	766,462	22.69
3	787,613	9.43	731,049	14.70	56,564	1.67
4	100,984	1.21	96,689	1.94	4295	0.13

^a^ Heavy drink: >2 drinks/day for men & > 1 drink/day for women (1 drink = 14 g alcohol).

#### 3.1.1 Effect of income

**FIGURE 2:**
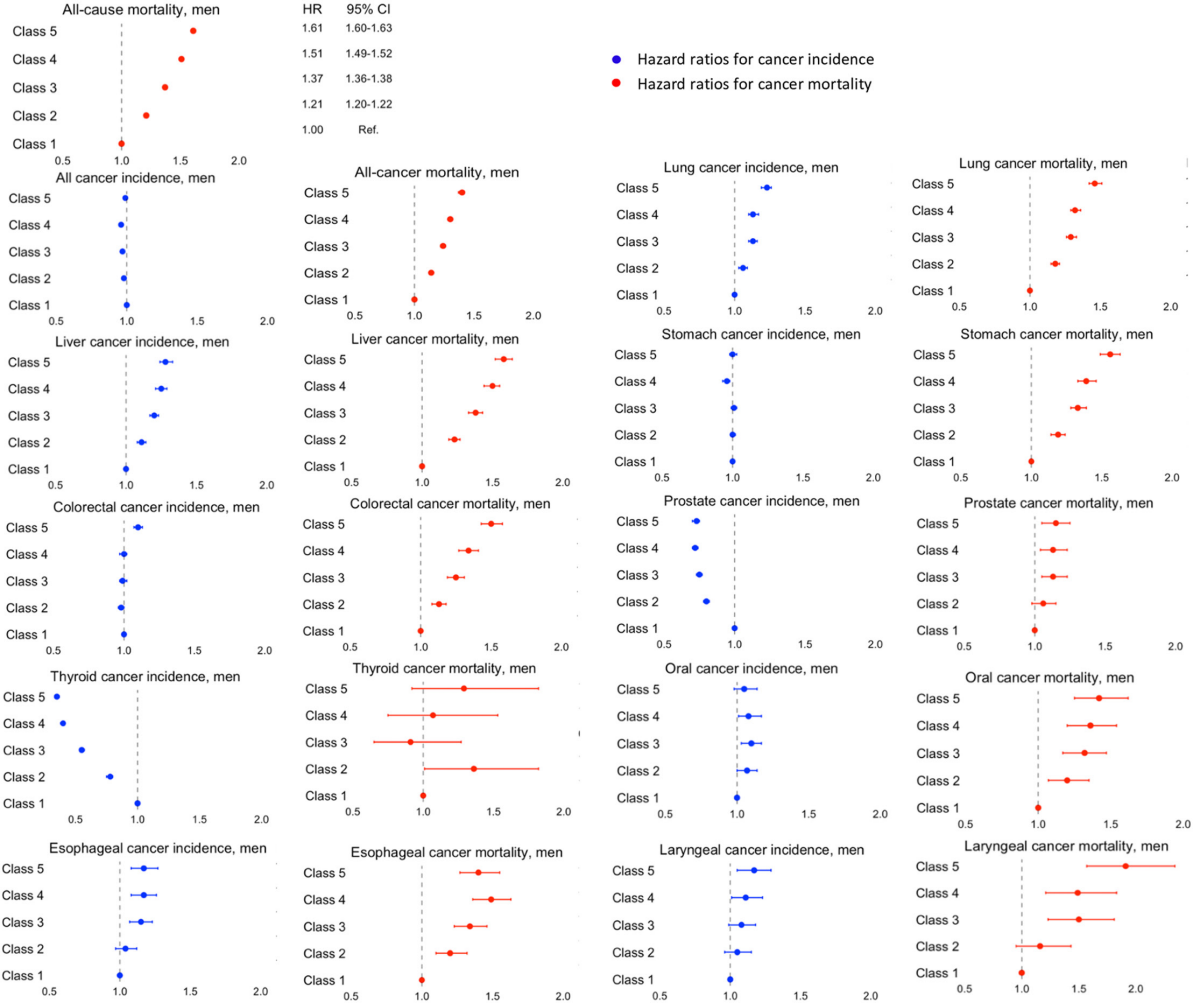
Effect of income on all‐cause mortality, cancer incidence, and cancer mortality among men. Hazard ratios are adjusted for age, smoking status, alcohol consumption, physical activity, BMI, CCI, area, and insurance subscriber types as well as chronic viral hepatitis for liver cancer when appropriate. Data are shown with error bars representing 95% confidence intervals of the hazard ratios.

Figure 2 shows the aHRs for the effect of income on cancer incidence, cancer‐specific mortality, and all‐cause mortality among men after adjusting for all covariates. Detailed results are shown in Tables S4 and S5. Low income was associated with a significantly higher incidence of lung, liver, esophageal, and laryngeal cancers but a lower incidence of prostate and thyroid cancers among men. Low income was also related to a significant increase in cancer‐specific mortality for most cancers except for thyroid cancer.

**FIGURE 3:**
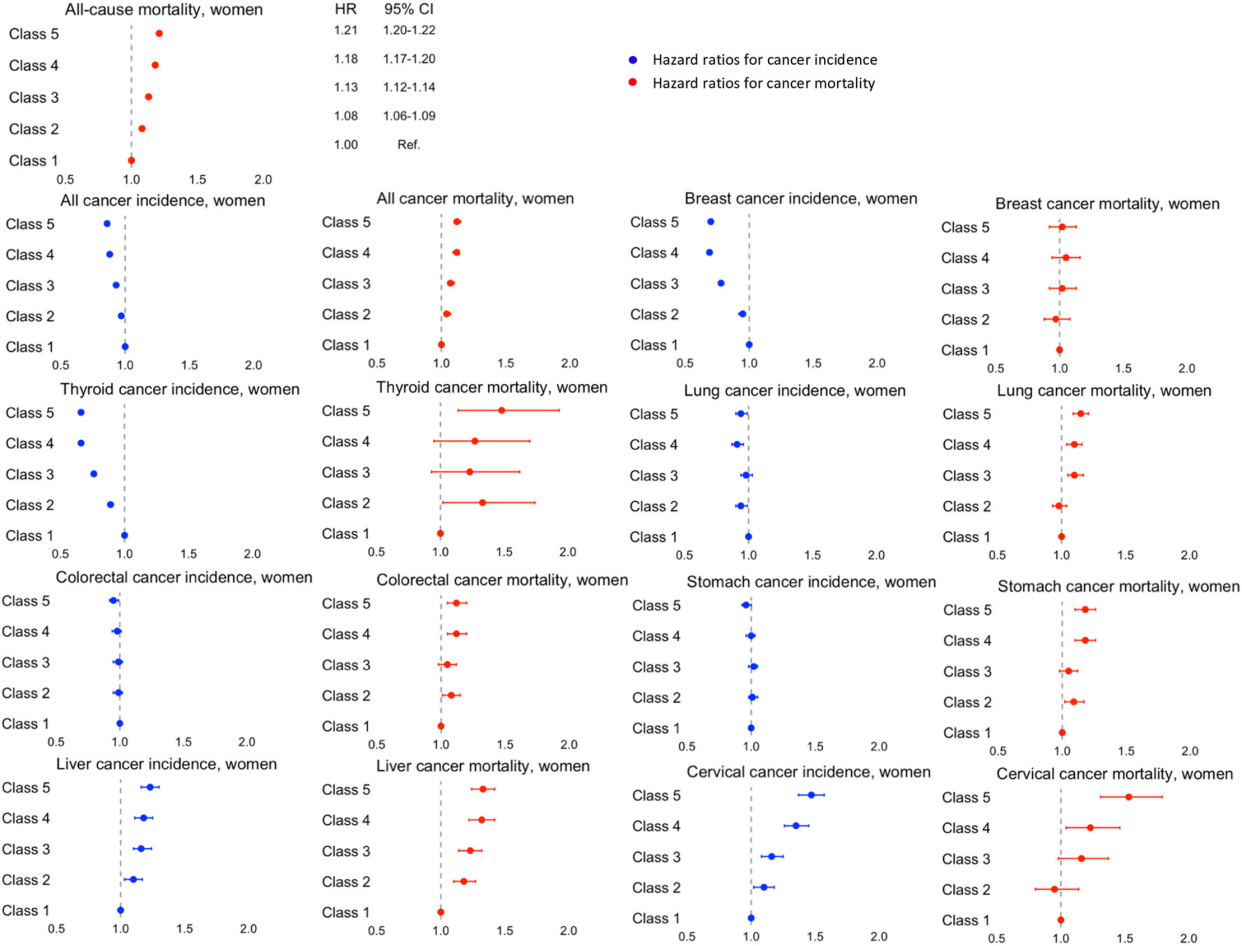
Effect of income on all‐cause mortality, cancer incidence, and cancer mortality among women. Hazard ratios are adjusted for age, smoking status, alcohol consumption, physical activity, BMI, CCI, area, and insurance subscriber types as well as chronic viral hepatitis for liver cancer when appropriate. Data are shown with error bars representing 95% confidence intervals of the hazard ratios.

Among women, low income was associated with a higher incidence of liver and cervical cancers but a lower incidence of cancer overall, and thyroid, breast, and lung cancers. An increased risk of cancer‐specific mortality corresponding to lower income levels in a gradient manner was also found, except for breast cancer (Figure 3 and Tables S6 and S7).

### 3.2 Effect of combined unhealthy lifestyle

Table 2 presents the aHRs for the effect of combined unhealthy lifestyle on cancer in men and women. Higher unhealthy lifestyle scores resulted in a dose–response increased risk of cancer incidence, cancer‐specific mortality, and all‐cause mortality in both sexes. Similar results were found for cancer incidence and cancer‐specific mortality for most cancer types, except for thyroid cancer in both sexes, prostate cancer in men, and breast cancer in women.

**TABLE 2:** Effect of combined unhealthy behaviors on cancer incidence, cancer mortality, and all‐cause mortality among men and women.

Unhealthy score	HRs^a^ (95% CI) among men					*p*‐trend^b^		HRs^a^ (95% CI) among women				*p*‐trend^b^
	0	1	2	3	4 (unhealthy)			0	1	2	≥3 (unhealthy)	
Cancer incidence							Cancer incidence					
All cancer	*Ref*.	1.16 (1.15–1.18)	1.32 (1.30–1.33)	1.47 (1.45–1.49)	1.61 (1.57–1.64)	<0.0001	All cancer	*Ref*.	0.99 (0.98–1.00)	1.02 (1.01–1.03)	1.09 (1.06–1.12)	<0.0001
Lung cancer	*Ref*.	1.68 (1.61–1.75)	2.27 (2.19–2.36)	2.68 (2.57–2.79)	2.91 (2.73–3.11)	<0.0001	Breast cancer	*Ref*.	0.93 (0.91–0.95)	0.91 (0.88–0.93)	0.86 (0.80–0.93)	<0.0001
Stomach cancer	*Ref*.	1.22 (1.19–1.26)	1.39 (1.36–1.43)	1.54 (1.50–1.59)	1.71 (1.63–1.80)	<0.0001	Colorectal cancer	*Ref*.	1.01 (0.98–1.05)	1.09 (1.05–1.12)	1.31 (1.20–1.42)	<0.0001
Colorectal cancer	*Ref*.	1.14 (1.11–1.18)	1.28 (1.24–1.32)	1.49 (1.44–1.54)	1.66 (1.57–1.76)	<0.0001	Stomach cancer	*Ref*.	1.02 (0.99–1.06)	1.04 (1.01–1.08)	1.17 (1.07–1.28)	0.0008
Liver cancer	*Ref*.	1.32 (1.27–1.38)	1.58 (1.52–1.64)	1.9 (1.82–1.98)	2.29 (2.14–2.45)	<0.0001	Lung cancer	*Ref*.	0.96 (0.92–1.00)	0.98 (0.94–1.03)	1.77 (1.61–1.94)	<0.0001
Oral cavity cancer	*Ref*.	1.2 (1.10–1.31)	1.44 (1.32–1.56)	1.74 (1.58–1.91)	2.13 (1.84–2.48)	<0.0001	Liver cancer	*Ref*.	1.09 (1.03–1.15)	1.3 (1.22–1.38)	1.64 (1.44–1.87)	<0.0001
Esophagus cancer	*Ref*.	1.52 (1.36–1.70)	2.19 (1.97–2.44)	3.45 (3.08–3.86)	4.61 (3.94–5.38)	<0.0001	Cervical cancer	*Ref*.	1.01 (0.96–1.07)	1.13 (1.06–1.21)	1.52 (1.32–1.75)	<0.0001
Larynx cancer	*Ref*.	2.02 (1.73–2.36)	2.76 (2.37–3.21)	3.86 (3.29–4.52)	4.41 (3.53–5.53)	<0.0001	Thyroid cancer	*Ref*.	0.94 (0.92–0.96)	0.91 (0.89–0.93)	0.67 (0.62–0.72)	<0.0001
Thyroid cancer	*Ref*.	0.97 (0.93–1.01)	1 (0.96–1.04)	1.01 (0.96–1.06)	0.91 (0.82–1.01)	0.4955						
Prostate cancer	*Ref*.	0.9 (0.88–0.93)	0.85 (0.83–0.88)	0.84 (0.81–0.87)	0.87 (0.80–0.94)	<0.0001						
Cancer mortality							Cancer mortality					
All cancer death	*Ref*.	1.3 (1.28–1.33)	1.6 (1.58–1.63)	1.87 (1.83–1.91)	2.11 (2.04–2.19)	<0.0001	All cancer death	*Ref*.	1.06 (1.04–1.09)	1.16 (1.14–1.19)	1.66 (1.58–1.74)	<0.0001
Lung cancer	*Ref*.	1.78 (1.71–1.86)	2.58 (2.48–2.69)	3.1 (2.97–3.24)	3.31 (3.09–3.54)	<0.0001	Breast cancer	*Ref*.	0.99 (0.91–1.07)	1.05 (0.96–1.16)	1.22 (0.96–1.55)	0.1235
Stomach cancer	*Ref*.	1.23 (1.17–1.30)	1.4 (1.33–1.48)	1.54 (1.45–1.63)	1.61 (1.45–1.78)	<0.0001	Colorectal cancer	*Ref*.	1.07 (1.00–1.14)	1.2 (1.13–1.29)	1.56 (1.35–1.80)	<0.0001
Colorectal cancer	*Ref*.	1.15 (1.09–1.22)	1.28 (1.21–1.36)	1.52 (1.42–1.62)	1.8 (1.61–2.02)	<0.0001	Stomach cancer	*Ref*.	1.08 (1.01–1.15)	1.07 (1.00–1.15)	1.39 (1.19–1.62)	0.0048
Liver cancer	*Ref*.	1.33 (1.27–1.40)	1.61 (1.54–1.69)	1.97 (1.88–2.07)	2.55 (2.36–2.75)	<0.0001	Lung cancer	*Ref*.	1.03 (0.98–1.09)	1.13 (1.07–1.19)	2.52 (2.29–2.77)	<0.0001
Oral cavity cancer	*Ref*.	1.25 (1.06–1.46)	1.66 (1.42–1.94)	2.16 (1.83–2.56)	2.55 (1.97–3.31)	<0.0001	Liver cancer	*Ref*.	1.16 (1.09–1.25)	1.41 (1.31–1.51)	2.01 (1.74–2.32)	<0.0001
Esophageal cancer	*Ref*.	1.48 (1.30–1.68)	2.12 (1.86–2.40)	3.24 (2.83–3.70)	4.21 (3.48–5.10)	<0.0001	Cervical cancer	*Ref*.	1.04 (0.90–1.20)	1.1 (0.94–1.30)	2.17 (1.61–2.91)	0.0022
Laryngeal cancer	*Ref*.	1.55 (1.15–2.09)	2.63 (1.97–3.50)	3.61 (2.66–4.89)	4.69 (3.05–7.20)	<0.0001	Thyroid cancer	*Ref*.	1.03 (0.80–1.34)	1.35 (1.03–1.77)	0.91 (0.44–1.89)	0.0299
Thyroid cancer	*Ref*.	1.34 (0.90–2.01)	1.53 (1.03–2.28)	1.54 (0.97–2.43)	1.21 (0.47–3.13)	0.076						
Prostate cancer	*Ref*.	1.11 (1.01–1.21)	1.21 (1.11–1.33)	1.31 (1.17–1.45)	1.32 (1.06–1.64)	<0.0001						
All‐cause mortality	*Ref*.	1.24 (1.22–1.25)	1.45 (1.43–1.46)	1.66 (1.64–1.68)	1.81 (1.77–1.85)	<0.0001	All‐cause mortality	*Ref*.	1.14 (1.13–1.15)	1.23 (1.21–1.24)	1.77 (1.73–1.81)	<0.0001

^a^ Hazard ratios adjusted for age, income, residential area, insurance subscriber types, CCI, and chronic viral hepatitis for liver cancer.

^b^ *p* for linear trend was calculated by modeling the combined score as a continuous variable.

### 3.3 Association of income with unhealthy lifestyle

The more deprived groups had higher odds of ever smoking, heavy alcohol consumption, physically inactive, and having extreme BMI. Lower income groups also had higher odds of having multiple unhealthy lifestyle factors than did the highest income group (Table S8).

### 3.4 Joint effect of income and unhealthy lifestyle on cancer

**FIGURE 4:**
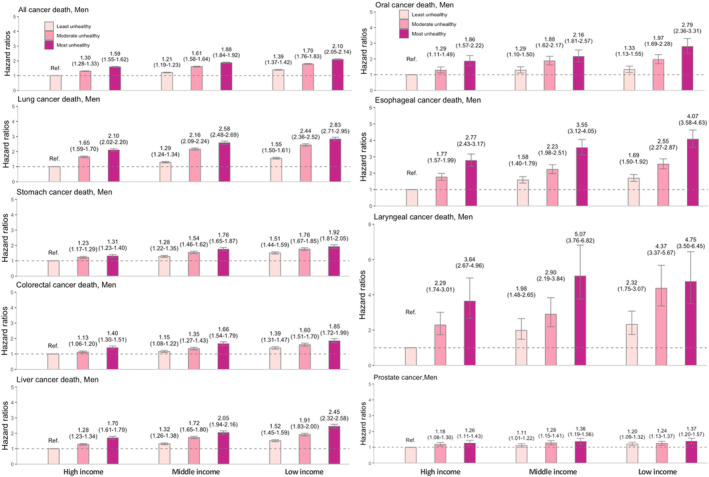
Joint association between income combined with unhealthy lifestyle and cancer mortality among men. Hazard ratios are adjusted for age, CCI, area, and insurance subscriber types as well as chronic viral hepatitis for liver cancer when appropriate. Data are shown with error bars representing 95% confidence intervals of the hazard ratios.

Figure 4 shows the joint effect of income and unhealthy lifestyle on cancer‐specific mortality among men. For all‐cancer death, a dose–response increase was observed for aHRs across all lifestyle categories and poverty levels when comparing to the combined healthiest and wealthiest reference group. The 95% CI of the aHR for each group did not overlap, indicating different risks of all‐cancer death. The most deprived individuals with unhealthiest behaviors experienced the highest risk (aHR = 2.10 [2.05–2.14]). For cancer‐specific mortality, the joint effect was most robust for head and neck cancer, with aHRs of 4.75 (3.50–6.45), 4.07 (3.58–4.63), and 2.79 (2.36–3.31) for laryngeal, esophageal, and oral cancer, respectively, followed by lung (aHR 2.83 [2.71–2.95]), liver (aHR 2.45 [2.32–2.58]), stomach (aHR 1.92 [1.81–2.05]), and colorectal cancers (aHR 1.85 [1.72–1.99]).

**FIGURE 5:**
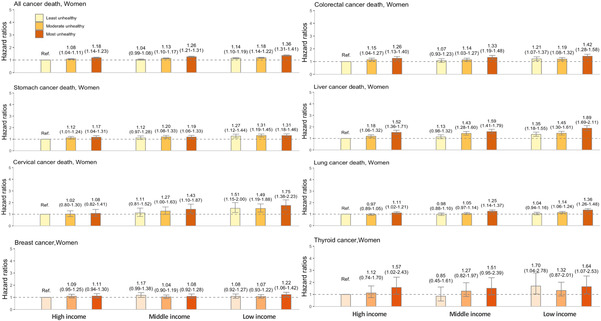
Joint association between income combined with unhealthy lifestyle and cancer mortality among women. Hazard ratios are adjusted for age, CCI, area, and insurance subscriber types as well as chronic viral hepatitis for liver cancer when appropriate. Data are shown with error bars representing 95% confidence intervals of the hazard ratios.

Figure 5 shows the joint effect on cancer‐specific mortality in women, with the results not being as substantial as those in men. For all‐cancer death, the risk among the unhealthiest and poorest women was 1.36‐fold higher (aHR = 1.36 [1.31–1.41]). For cancer‐specific mortality, the strongest effect was observed in liver (aHR 1.89 [1.69–2.11]) and cervical cancers (aHR 1.75 [1.38–2.23]).

We also observed the joint effect of income and unhealthy lifestyle on the incidence of lung, liver, oral cavity, esophageal, and laryngeal cancers in men and liver and cervical cancers in women (Figures S2 and S3). The joint association was stronger in men than in women.

For all‐cause mortality, the lowest income and unhealthiest men had a 2.24‐fold higher risk (aHR = 2.24 [2.22–2.27]), while women in these groups showed a 1.54‐fold higher risk (aHR = 1.54 [1.52–1.58]) (Figure S4) than their corresponding reference groups.

## 4 DISCUSSION

We found that low income was associated with an elevated risk that followed a social gradient for all‐cause mortality, all‐cancer death, and death due to most cancers. Low income appeared to have a varied association with cancer incidence depending on cancer type in both sexes. Combined unhealthy lifestyles showed a dose–response increased risk of all‐cause mortality, cancer incidence, and cancer‐specific mortality for most cancers; in addition, lower income groups were more likely to engage in multiple unhealthy behaviors. Individuals with the lowest income and unhealthiest lifestyles were most vulnerable to all‐cause mortality, cancer incidence, and mortality from several cancers, and this joint effect appeared to be stronger with cancer‐specific mortality than with cancer incidence, and stronger in men than in women.

Overall, the effect of income on health outcomes is relatively consistent with the findings of previous studies.^30^, ^33^ The effect of income on incidence varied by cancer type: those highly related to modifiable risk factors, including smoking (lung cancer in men), heavy alcohol consumption (head and neck cancer), and infections (hepatitis virus for liver cancer and human papillomavirus [HPV] for cervical cancer) showed a significantly higher incidence among the poor. The unequal distribution of unhealthy lifestyle risk factors among income groups may contribute to social inequalities in these cancers.^1^ After adjusting for these modifiable risk factors, the results were attenuated but still consistent. In contrast, cancers that may involve over‐screening and overdiagnosis, including thyroid and breast cancers in women and prostate cancer in men, showed a higher incidence among the rich,^1^ possibly due to better accessibility to screening. One study revealed that Korean women with higher household incomes had more frequent thyroid cancer screening^42^; another identified inequalities in breast cancer screening among Korean women from 2005 to 2015.^43^ A Swedish study also found that higher income men had a higher probability of detecting prostate cancer during routine check‐ups.^44^ However, the lower income group showed a higher cancer‐specific mortality rate for most cancers, which could result from poorer access to screening and appropriate treatment, leading to a higher proportion of late‐stage cases.^45^, ^46^

We found that individuals with unhealthy lifestyles had poorer health outcomes, as in previous studies.^27^, ^28^ Multiple unhealthy behaviors (including smoking, alcohol drinking, physical inactivity, unhealthy diet, abnormal sleep duration, and television viewing time) were found to be strongly associated with CVDs and all‐cause mortality^28^; in addition, healthy lifestyle (non‐smoking, non‐drinking, physical activity, maintaining a healthy diet, and a waist‐to‐hip ratio <0.90 for men and <0.85 for women) reduced liver cancer risk by 43% in the Chinese.^27^ However, one study found no significant association between multiple unhealthy behaviors and cancer‐specific death among Koreans.^47^ This difference could be due to the shorter follow‐up time (average: 6.01 years) in their study than in ours (average: 16.5 years).

For breast and thyroid cancers in women and prostate cancer in men, we found a lower risk of incidence among unhealthy individuals, possibly due to less participation in healthcare services such as cancer screening. It was found that people with multiple unhealthy behaviors were less likely to visit a general practitioner^48^; additionally, Chinese people who habitually smoked, drank alcohol, and were physically inactive had poor health‐seeking behavior.^49^ In addition, it was suggested that obese Korean women are less likely to follow recommended screening schedules for breast and cervical cancers,^50^ and smoking was found to be associated with a lower rate of breast cancer screening among Korean women.^51^

We found that the poorest people with unhealthiest lifestyles were most vulnerable to all‐cause death, cancer‐related death, and several cancers. Here, the increase in all‐cause mortality was 2.24‐fold (2.22–2.27) among men and 1.54‐fold (1.52–1.58) among women, which is smaller than in previous Western studies. In the United States, adults with low SES and the unhealthiest lifestyles had a 3.53‐fold (3.01–4.14) higher risk of all‐cause death than those with high SES and healthy lifestyles; in the United Kingdom, the increase was 2.65‐fold (2.39–2.94).^29^ This might be due to differences in SES assessment, lifestyle factors, source population, and health policies.

We observed a stronger joint effect of SES and lifestyle factors on cancer‐specific mortality than on cancer incidence. This could be because the discrepancy in cancer‐specific mortality is driven not only by the inequality in cancer incidence and related causes but also by factors such as late diagnosis and poorer access to treatment among low‐SES groups.^5^, ^11^, ^46^ Koreans of the lowest SES were more likely to be diagnosed at later stages for stomach, colorectal, and female breast cancer^46^; other studies have also shown higher odds for cervical (OR 1.46 [1.28–1.67])^52^ and breast cancer (OR 2.25 [1.97–2.58]).^53^ In addition, low SES is associated with poorer access to cancer care, healthcare delay, and lower‐quality treatment,^54^, ^55^ with high‐income patients using healthcare services more frequently and mostly at tertiary hospitals with better medical care compared to low‐income patients.^55^

These findings imply that cancer‐specific mortality could be a more meaningful endpoint than cancer incidence for investigating cancer disparities. Additionally, more comprehensive strategies are required to reduce inequalities in cancer care, such as by increasing the screening rate among low‐income groups. Participants in cancer screening had higher age‐standardized prevalence rates of early‐stage cancer by income group than that of non‐participants.^56^ In addition, improving cancer awareness and access to appropriate and quality treatment for marginalized populations could reduce inequalities in cancer care. The Financial Aid Program for Cancer Patients (FAPCP), a government initiative to provide financial aid to patients with cancer, was established in Korea in 2002. However, 87.6% of FAPCP recipients still reported significant financial burdens, and 10.2% changed or discontinued treatment because of medical expenses.^57^ Therefore, the FAPCP should broaden its benefits and support services, especially for the most vulnerable cancer patients, to help minimize these inequalities. In addition, policies should target the social determinants of health and promote lifestyle adjustment particularly among disadvantaged groups to improve population health and minimize social disparities.

This study has several limitations. First, our study lacked information on education level and occupation to assess overall SES. Second, our data lacked information on diet, a risk factor for several malignancies. We also lacked information on other risk factors, including secondary smoking, reproductive factors, *Helicobacter pylori* and HPV infection, genetic factors, and family history of specific cancers. Third, our unhealthy lifestyle score implies that each factor has equal risk for each outcome; Hence, further studies using weighted scores specific to each cancer outcome are needed. Fourth, we lacked information regarding cancer screening and stages to better comprehend social inequalities in cancer. Fifth, due to only including participants who received health examinations (i.e., 30% of the eligible population),^58^ this cohort study was unable to represent the entire Korean population. Finally, SES and lifestyle behaviors may have changed over time, affecting the results. Therefore, the effect of income trajectory on health could be a topic for future studies. Income can change over time during a person's life, and the effect on health might vary largely due to the increase or decrease trend of income. Several studies on income fluctuations suggested that long‐term income might have a stronger relation to health than baseline income.^59^, ^60^

In conclusion, our study proved that income and combined healthy lifestyle behaviors (including smoking, heavy alcohol drinking, being physically inactive, and having an unhealthy BMI) were independently associated with a significantly increased risk of all‐cause mortality, cancer mortality, and cancer incidence of several cancers. The effect on cancer‐specific incidence and mortality varies by sex and cancer type. We also found that lower income groups have higher odds of having multiple unhealthy behaviors compared to the highest income group. In addition, the combined effect of income and unhealthy lifestyle results in an elevated risk of health outcomes, with individuals of the lowest income and the unhealthiest lifestyle experiencing the highest risk of all‐cause mortality, cancer mortality, and cancer incidence of several cancer types. These results suggest that policies should both target social determinants of health and promote lifestyle adjustment among the population, particularly among disadvantaged groups, in order to improve population health and minimize social health disparities.

## AUTHOR CONTRIBUTIONS

**Chi Lan Tran:** Conceptualization (equal); formal analysis (lead); investigation (equal); methodology (lead); writing – original draft (lead). **Kui Son Choi:** Writing – review and editing (equal). **Sun young Kim:** Writing – review and editing (equal). **Jin‐Kyoung Oh:** Conceptualization (equal); investigation (equal); writing – review and editing (equal).

## FUNDING INFORMATION

This work was supported by the National Cancer Center (grant number: NCC‐2210862). The funder had no role in the study design, data collection, analysis, decision to publish, or preparation of the manuscript.

## CONFLICT OF INTEREST STATEMENT

The authors declare no conflict of interest.

## ETHICS STATEMENT

Using anonymous secondary data, this study was exempted from review by the Institutional Review Board of the National Cancer Center, Korea (NCC2022‐0180).

## CONSENT STATEMENT

Informed consent was waived for the same reason.

## Supporting information


Appendix S1.

## Data Availability

The datasets generated and analyzed during the current study cannot be shared because NHIS prohibits the transfer, rental, or sale of the database to third parties except for researchers who have been approved for access. NHIS data are available upon request from the National Health Insurance Sharing Service, https://nhiss.nhis.or.kr/.

## References

[1] Vaccarella SL‐TJ , Saracci R , Conway DI , Straif K , Wild CP . Reducing social inequalities in cancer: evidence and priorities for research. The International Agency for Research on Cancer; 2019. 33443989

[2] Sharpe KH , McMahon AD , Raab GM , Brewster DH , Conway DI . Association between socioeconomic factors and cancer risk: a population cohort study in Scotland (1991‐2006). PloS One. 2014;9:e89513. 24586838 10.1371/journal.pone.0089513 PMC3937337

[3] Singh GK , Jemal A . Socioeconomic and racial/ethnic disparities in cancer mortality, incidence, and survival in the United States, 1950‐2014: over six decades of changing patterns and widening inequalities. J Environ Public Health. 2017;2017:2819372. 28408935 10.1155/2017/2819372 PMC5376950

[4] Larsen IK , Myklebust TA , Babigumira R , Vinberg E , Moller B , Ursin G . Education, income and risk of cancer: results from a Norwegian registry‐based study. Acta Oncol. 2020;59:1300‐1307. 32924698 10.1080/0284186X.2020.1817548

[5] Rosskamp M , Verbeeck J , Gadeyne S , Verdoodt F , De Schutter H . Socio‐economic position, cancer incidence and stage at diagnosis: a Nationwide cohort study in Belgium. Cancers (Basel). 2021;13:13. 10.3390/cancers13050933 PMC7956180 33668089

[6] Bryere J , Dejardin O , Launay L , et al. Socioeconomic status and site‐specific cancer incidence, a Bayesian approach in a French cancer registries Network study. Eur J Cancer Prev. 2018;27:391‐398. 27879493 10.1097/CEJ.0000000000000326

[7] Conway DI , Brenner DR , McMahon AD , et al. Estimating and explaining the effect of education and income on head and neck cancer risk: INHANCE consortium pooled analysis of 31 case‐control studies from 27 countries. Int J Cancer. 2015;136:1125‐1139. 24996155 10.1002/ijc.29063 PMC4531373

[8] Lagergren J , Andersson G , Talback M , et al. Marital status, education, and income in relation to the risk of esophageal and gastric cancer by histological type and site. Cancer. 2016;122:207‐212. 26447737 10.1002/cncr.29731

[9] Vaccarella S , Franceschi S , Bray F , Wild CP , Plummer M , Dal Maso L . Worldwide thyroid‐cancer epidemic? The increasing impact of Overdiagnosis. N Engl J Med. 2016;375:614‐617. 27532827 10.1056/NEJMp1604412

[10] Martin RM , Donovan JL , Turner EL , et al. Effect of a low‐intensity PSA‐based screening intervention on prostate cancer mortality: the CAP randomized clinical trial. JAMA. 2018;319:883‐895. 29509864 10.1001/jama.2018.0154 PMC5885905

[11] Boscoe FP , Henry KA , Sherman RL , Johnson CJ . The relationship between cancer incidence, stage and poverty in the United States. Int J Cancer. 2016;139:607‐612. 26991033 10.1002/ijc.30087

[12] Lee K , Lim HT , Hwang SS , Chae DW , Park SM . Socio‐economic disparities in behavioural risk factors for cancer and use of cancer screening services in Korean adults aged 30 years and older: the third Korean National Health and nutrition examination survey, 2005 (KNHANES III). Public Health. 2010;124:698‐704. 20888016 10.1016/j.puhe.2010.07.004

[13] Wild CP , Weiderpass E , Stewart BW . World Cancer Report: Cancer Research for Cancer Prevention. 2020. http://publications.iarc.fr/586 39432694

[14] Lushniak BD , Samet JM , Pechacek TF , Norman LA , Taylor PA . The health consequences of smoking—50 years of progress: a report of the surgeon general. Report. 2014.

[15] WCRF/AICR . Diet, Nutrition, Physical Activity, and Cancer: A Global Perspective. Accessed September 14, 2022. https://www.wcrf.org/diet‐activity‐and‐cancer/

[16] Rock CL , Thomson C , Gansler T , et al. American Cancer Society guideline for diet and physical activity for cancer prevention. CA Cancer J Clin. 2020;70:245‐271. 32515498 10.3322/caac.21591

[17] McTiernan A , Friedenreich CM , Katzmarzyk PT , et al. Physical activity in cancer prevention and survival: a systematic review. Med Sci Sports Exerc. 2019;51:1252‐1261. 31095082 10.1249/MSS.0000000000001937 PMC6527123

[18] Kraus WE , Powell KE , Haskell WL , et al. Physical activity, all‐cause and cardiovascular mortality, and cardiovascular disease. Med Sci Sports Exerc. 2019;51:1270‐1281. 31095084 10.1249/MSS.0000000000001939 PMC6527136

[19] Zhang YB , Pan XF , Chen J , et al. Combined lifestyle factors, incident cancer, and cancer mortality: a systematic review and meta‐analysis of prospective cohort studies. Br J Cancer. 2020;122:1085‐1093. 32037402 10.1038/s41416-020-0741-x PMC7109112

[20] Arthur R , Wassertheil‐Smoller S , Manson JE , et al. The combined Association of Modifiable Risk Factors with breast cancer risk in the Women's Health Initiative. Cancer Prev Res (Phila). 2018;11:317‐326. 29483073 10.1158/1940-6207.CAPR-17-0347 PMC6866659

[21] Chen SLF , Braaten T , Borch KB , Ferrari P , Sandanger TM , Nost TH . Combined lifestyle behaviors and the incidence of common cancer types in the Norwegian women and cancer study (NOWAC). Clin Epidemiol. 2021;13:721‐734. 34429658 10.2147/CLEP.S312864 PMC8378914

[22] Freisling H , Viallon V , Lennon H , et al. Lifestyle factors and risk of multimorbidity of cancer and cardiometabolic diseases: a multinational cohort study. BMC Med. 2020;18:5. 31918762 10.1186/s12916-019-1474-7 PMC6953215

[23] McKenzie F , Biessy C , Ferrari P , et al. Healthy lifestyle and risk of cancer in the European prospective investigation into cancer and nutrition cohort study. Medicine (Baltimore). 2016;95:e2850. 27100409 10.1097/MD.0000000000002850 PMC4845813

[24] Wang K , Ma W , Wu K , et al. Healthy lifestyle, endoscopic screening, and colorectal cancer incidence and mortality in the United States: a nationwide cohort study. PLoS Med. 2021;18:e1003522. 33524029 10.1371/journal.pmed.1003522 PMC7886195

[25] Korn AR , Reedy J , Brockton NT , Kahle LL , Mitrou P , Shams‐White MM . The 2018 World Cancer Research Fund/American Institute for Cancer Research score and cancer risk: a longitudinal analysis in the NIH‐AARP diet and health study. Cancer Epidemiol Biomarkers Prev. 2022;31:1983‐1992. 35877953 10.1158/1055-9965.EPI-22-0044 PMC9532348

[26] Luu HN , Behari J , Goh GB , et al. Composite score of healthy lifestyle factors and risk of hepatocellular carcinoma: findings from a prospective cohort study. Cancer Epidemiol Biomarkers Prev. 2021;30:380‐387. 33187965 10.1158/1055-9965.EPI-20-1201 PMC7867589

[27] Song C , Lv J , Yu C , et al. Adherence to healthy lifestyle and liver cancer in Chinese: a prospective cohort study of 0.5 million people. Br J Cancer. 2022;126:815‐821. 34853434 10.1038/s41416-021-01645-x PMC8888610

[28] Foster HME , Celis‐Morales CA , Nicholl BI , et al. The effect of socioeconomic deprivation on the association between an extended measurement of unhealthy lifestyle factors and health outcomes: a prospective analysis of the UK biobank cohort. Lancet Public Health. 2018;3:e576‐e585. 30467019 10.1016/S2468-2667(18)30200-7

[29] Zhang YB , Chen C , Pan XF , et al. Associations of healthy lifestyle and socioeconomic status with mortality and incident cardiovascular disease: two prospective cohort studies. BMJ. 2021;373:n604. 33853828 10.1136/bmj.n604 PMC8044922

[30] Kim JM , Kim HM , Jung BY , Park EC , Cho WH , Lee SG . The association between cancer incidence and family income: analysis of Korean National Health Insurance cancer registration data. Asian Pac J Cancer Prev. 2012;13:1371‐1376. 22799334 10.7314/apjcp.2012.13.4.1371

[31] Choi SW , Ryu SY , Han MA , Park J . The association between the socioeconomic status and thyroid cancer prevalence; based on the Korean National Health and nutrition examination survey 2010‐2011. J Korean Med Sci. 2013;28:1734‐1740. 24339702 10.3346/jkms.2013.28.12.1734 PMC3857368

[32] Hur HW , Ryu SY , Park J , Choi SW . Relationship between socioeconomic status and prevalent prostate cancer in the South Korea. Asian Pac J Cancer Prev. 2019;20:3137‐3144. 31653165 10.31557/APJCP.2019.20.10.3137 PMC6982686

[33] Kim C‐W , Lee S‐Y , Moon O‐R . Inequalities in cancer incidence and mortality across income groups and policy implications in South Korea. Public Health. 2008;122:229‐236. 17935744 10.1016/j.puhe.2007.07.003

[34] Oh JK , Han M , Kim B , Park EY . Adherence to cancer prevention guidelines and cancer incidence and mortality: a population‐based cohort study. Cancer Res Treat. 2022;55(1):15‐27. 35344651 10.4143/crt.2021.1031 PMC9873327

[35] Lee J , Lee JS , Park S‐H , Shin SA , Kim K . Cohort profile: the National Health Insurance Service–National Sample Cohort (NHIS‐NSC), South Korea. Int J Epidemiol. 2016;46:e15. 10.1093/ije/dyv319 26822938

[36] Kyoung DS , Kim HS . Understanding and utilizing claim data from the Korean National Health Insurance Service (NHIS) and Health Insurance Review & Assessment (HIRA) database for research. J Lipid Atheroscler. 2022;11:103‐110. 35656154 10.12997/jla.2022.11.2.103 PMC9133780

[37] Yang MS , Park M , Back JH , et al. Validation of cancer diagnosis based on the National Health Insurance Service database versus the National Cancer Registry Database in Korea. Cancer Res Treat. 2022;54:352‐361. 34353000 10.4143/crt.2021.044 PMC9016317

[38] Kang MJ , Won YJ , Lee JJ , et al. Community of Population‐Based Regional Cancer R. Cancer statistics in Korea: incidence, mortality, survival, and prevalence in 2019. Cancer Res Treat. 2022;54:330‐344. 35313102 10.4143/crt.2022.128 PMC9016309

[39] Hsiao JR , Huang CC , Ou CY , et al. Investigating the health disparities in the association between lifestyle behaviors and the risk of head and neck cancer. Cancer Sci. 2020;111:2974‐2986. 32539207 10.1111/cas.14530 PMC7419018

[40] Bahk J , Kang HY , Khang YH . Inequality in life expectancy in Korea according to various categorizations of the National Health Insurance Premiums as a marker of income. Yonsei Med J. 2020;61:640‐643. 32608209 10.3349/ymj.2020.61.7.640 PMC7329743

[41] Inoue S , Zimmet P , Caterson I , et al. The Asia‐Pacific Perspective: Redefining Obesity and its Treatment. World Health Organization Western Pacific Region/International Association for the Study of Obesity; 2000.

[42] Cho HN , Choi E , Seo DH , et al. Determinants of undergoing thyroid cancer screening in Korean women: a cross‐sectional analysis from the K‐Stori 2016. BMJ Open. 2019;9:e026366. 10.1136/bmjopen-2018-026366 PMC6500224 30948602

[43] Choi E , Lee YY , Suh M , et al. Socioeconomic inequalities in cervical and breast cancer screening among women in Korea, 2005‐2015. Yonsei Med J. 2018;59:1026‐1033. 30328316 10.3349/ymj.2018.59.9.1026 PMC6192888

[44] Tomic K , Ventimiglia E , Robinson D , Haggstrom C , Lambe M , Stattin P . Socioeconomic status and diagnosis, treatment, and mortality in men with prostate cancer. Nationwide population‐based study. Int J Cancer. 2018;142:2478‐2484. 29363113 10.1002/ijc.31272 PMC5947133

[45] Rajaguru V , Kim TH , Shin J , Lee SG . Income disparities in cancer screening: a cross‐sectional study of the Korean National Health and nutrition examination survey, 2013‐2019. Front Public Health. 2022;10:820643. 35372248 10.3389/fpubh.2022.820643 PMC8968859

[46] Kweon SS , Kim MG , Kang MR , Shin MH , Choi JS . Difference of stage at cancer diagnosis by socioeconomic status for four target cancers of the National Cancer Screening Program in Korea: results from the Gwangju and Jeonnam cancer registries. J Epidemiol. 2017;27:299‐304. 28279589 10.1016/j.je.2016.07.004 PMC5498418

[47] Lee DH , Nam JY , Kwon S , et al. Lifestyle risk score and mortality in Korean adults: a population‐based cohort study. Sci Rep. 2020;10:10260. 32581249 10.1038/s41598-020-66742-y PMC7314763

[48] Feng X , Girosi F , McRae I . People with multiple unhealthy lifestyles are less likely to consult primary healthcare. BMC Fam Pract. 2014;15:126. 24965672 10.1186/1471-2296-15-126 PMC4083035

[49] Li C , Sun J . The impact of current smoking, regular drinking, and physical inactivity on health care‐seeking behavior in China. BMC Health Serv Res. 2022;22:52. 35012543 10.1186/s12913-022-07462-z PMC8751354

[50] Park JK , Park HA , Park JJ , Cho YG . Obesity and screening compliance for breast and cervical cancer in Korean women. Asian Pac J Cancer Prev. 2012;13:3271‐3274. 22994746 10.7314/apjcp.2012.13.7.3271

[51] Lee K , Lim HT , Park SM . Factors associated with use of breast cancer screening services by women aged >or= 40 years in Korea: the third Korea National Health and nutrition examination survey 2005 (KNHANES III). BMC Cancer. 2010;10:144. 20398358 10.1186/1471-2407-10-144 PMC2873386

[52] Bolormaa E , Choe S‐A , Son M , Ki M , Paek D . Income‐based disparity in the risk distant‐stage cervical cancer and mortality after introduction of a national cancer screening program. Epidemiol Health. 2022;44:e2022066. 35989657 10.4178/epih.e2022066 PMC10089710

[53] Choe SA , Roh M , Kim HR , et al. Income disparity in breast cancer incidence and stage at presentation: a National Population Study of South Korea. J Breast Cancer. 2022;25:415‐424. 36265886 10.4048/jbc.2022.25.e38 PMC9629970

[54] Chang CM , Yin WY , Wei CK , et al. The association of socioeconomic status and access to low‐volume service providers in breast cancer. PloS One. 2013;8:e81801. 24312589 10.1371/journal.pone.0081801 PMC3846901

[55] Yoon TH , Lee S‐Y , Kim C‐W , Kim SY , Jeong B‐G , Park H‐K . Inequalities in medical care utilization by south Korean cancer patients according to income: a retrospective cohort study. Int J Health Serv. 2011;41:51‐66. 21319720 10.2190/HS.41.1.d

[56] Jung H‐M , Lee J‐S , Lairson DR , Kim Y . The effect of National Cancer Screening on disparity reduction in cancer stage at diagnosis by income level. PloS One. 2015;10:e0136036. 26284526 10.1371/journal.pone.0136036 PMC4540564

[57] Min HS , Yang HK , Park K . Supporting low‐income cancer patients: recommendations for the public financial aid program in the Republic of Korea. Cancer Res Treat. 2018;50:1074‐1083. 29141397 10.4143/crt.2017.401 PMC6192922

[58] Seong SC , Kim YY , Park SK , et al. Cohort profile: the National Health Insurance Service‐National Health Screening Cohort (NHIS‐HEALS) in Korea. BMJ Open. 2017;7:e016640. 10.1136/bmjopen-2017-016640 PMC5623538 28947447

[59] Bævre K , Kravdal Ø . The effects of earlier income variation on mortality: an analysis of Norwegian register data. Popul Stud (Camb). 2014;68:81‐94. 24134548 10.1080/00324728.2013.824603

[60] Veenstra M , Aartsen M . Life‐course income trajectories of men and women in Norway: implications for self‐rated health in later life. Eur J Public Health. 2022;32:542‐547. 35708604 10.1093/eurpub/ckac055 PMC9341848

