# Supplementary material for: Individual and joint effect of socioeconomic status and lifestyle factors on cancer in Korea
Source: Cancer Med. 2023 Jul 25;12(16):17389–402. doi: 10.1002/cam4.6359 (PMC10501257; doi:10.1002/cam4.6359)
Supplement: Supplementary file 1 — Appendix S1. [file CAM4-12-17389-s001.pdf]

SUPPLEMENT TABLES AND FIGURES

Supplement table 1. Characteristic of men by income classes

Income classes	Class 5 (low)		Class 4		Class 3		Class 2		Class 1 (high)	
	N	%	N	%	N	%	N	%	N	%
**Age**										
20-39	249809	46.05	432797	57.29	713732	57.41	554794	48.3	323696	25.19
40-65	244069	44.99	269049	35.61	466594	37.53	527621	45.94	864414	67.27
65+	48574	8.95	53596	7.09	62925	5.06	66182	5.76	96969	7.55
**Smoking status**										
Never	160776	29.64	212879	28.18	352313	28.34	348238	30.32	445593	34.67
Former	62510	11.52	90762	12.01	172711	13.89	191132	16.64	253755	19.75
Current	319166	58.84	451801	59.81	718227	57.77	609227	53.04	585731	45.58
**Alcohol consumption**										
Never	158944	29.3	194097	25.69	292066	23.49	264492	23.03	312618	24.33
Light drink	293278	54.07	431783	57.16	740049	59.53	698308	60.8	779387	60.65
Heavy drink	90230	16.63	129562	17.15	211136	16.98	185797	16.18	193074	15.02
**BMI**										
<18.5	16606	3.06	20613	2.73	24442	1.97	17895	1.56	17062	1.33
18.5-24.9	338749	62.45	478669	63.36	750591	60.37	669725	58.31	738150	57.44
25.0-29.9	168398	31.04	230636	30.53	424990	34.18	423711	36.89	494017	38.44
>=30	18699	3.45	25524	3.38	43228	3.48	37266	3.24	35850	2.79
**Physical activity**										
Do not exercise	223943	41.28	318357	42.14	492373	39.6	417573	36.36	411036	31.99
**Chronic hepatitis virus**	21679	4.00	32537	4.31	57303	4.61	55929	4.87	64881	5.05
**Insurance subscriber**										
Employment subscriber	464114	85.55	592618	78.45	991287	79.73	871125	75.84	930960	72.45
Local subscriber	76062	14.02	161052	21.32	251115	20.2	277160	24.13	354008	27.54
Medical benefit	2276	0.42	1772	0.23	849	0.07	312	0.03	111	0.01
**Area**										
Metropolitan city	259917	47.92	354492	46.93	589115	47.39	545811	47.52	665977	51.82
Province	282535	52.08	400950	53.07	654136	52.61	602786	52.48	619102	48.18
**Unhealthy lifestyle scores**										
0	48344	8.91	64007	8.47	109230	8.79	112482	9.79	154014	11.98
1	193804	35.73	266037	35.22	429922	34.58	404279	35.2	476833	37.11
2	206531	38.07	291452	38.58	482170	38.78	439281	38.25	468697	36.47
3	82406	15.19	117470	15.55	194871	15.67	170460	14.84	165842	12.91
4	11367	2.10	16476	2.18	27058	2.18	22095	1.92	19693	1.53

Supplement table 2. Characteristic of women by income classes

Income classes	Class 5 (low)		Class 4		Class 3		Class 2		Class 1 (high)	
	N	%	N	%	N	%	N	%	N	%
**Age**										
20-39	264576	38.44	295033	45.27	307660	43.05	182749	30	80729	11.3
40-65	352809	51.26	297593	45.66	342109	47.87	354371	58.18	524193	73.35
65+	70850	10.29	59066	9.06	64877	9.08	71978	11.82	109755	15.36
**Smoking status**										
Never	644326	93.62	608797	93.42	678795	94.98	585143	96.07	690613	96.63
Former	14686	2.13	14417	2.21	13053	1.83	8704	1.43	9383	1.31
Current	29223	4.25	28478	4.37	22798	3.19	15251	2.5	14681	2.05
**Alcohol consumption**										
Never	445861	64.78	414073	63.54	488535	68.36	458291	75.24	574104	80.33
Light drink	215012	31.24	211990	32.53	205978	28.82	137719	22.61	127783	17.88
Heavy drink	27362	3.98	25629	3.93	20133	2.82	13088	2.15	12790	1.79
**BMI**										
<18.5	39828	5.79	45895	7.04	45389	6.35	26051	4.28	21036	2.94
18.5-24.9	454542	66.04	439978	67.51	484421	67.78	410464	67.39	473511	66.26
25.0-29.9	169983	24.7	145094	22.26	162972	22.8	153589	25.22	198566	27.78
>=30	23882	3.47	20725	3.18	21864	3.06	18994	3.12	21564	3.02
**Physical activity**										
Do not exercise	436201	63.38	417886	64.12	450143	62.99	365992	60.09	381894	53.44
**Chronic hepatitis virus**	23581	3.43	22463	3.45	25087	3.51	23132	3.80	29282	4.10
**Insurance subscriber types**										
Employment subscriber	527823	76.69	454803	69.78	471487	65.98	344091	56.49	357245	49.98
Local subscriber	156106	22.68	194291	29.81	242120	33.88	264568	43.44	357236	49.98
Medical benefit	4306	0.62	2598	0.40	1039	0.14	439	0.07	196	0.02
**Area**										
Metropolitan city	315360	45.82	309135	47.44	338517	47.37	282937	46.45	359367	50.28
Province	372875	54.18	342557	52.56	376129	52.63	326161	53.55	355310	49.72
**Unhealthy lifestyle scores**										
0	149113	21.67	143355	22.00	168240	23.54	157745	25.9	215146	30.10
1	355371	51.64	335526	51.49	369787	51.74	309730	50.85	347014	48.56
2	166789	24.23	157065	24.10	164157	22.97	133377	21.9	145074	20.30
≥3	16962	2.46	15746	2.42	12462	1.74	8246	1.35	7443	1.04

Supplement table 3. Health outcomes among study participants

Outcomes	Total 8,353,169		Men 4,974,821		Women 3,378,348	
	N	%	N	%	N	%
**All-cause mortality**	734148	8.79	475162	9.55	258986	7.67
**Cancer mortality**						
All cancer death	236761	2.83	167331	3.36	69430	2.06
Colorectal cancer	22946	0.27	14798	0.30	8148	0.24
Stomach cancer	28359	0.34	20757	0.42	7602	0.23
Lung cancer	58367	0.70	46520	0.94	11847	0.35
Breast cancer	3635	0.04	52	0.00	3583	0.11
Liver cancer	35339	0.42	28074	0.56	7265	0.22
Prostate cancer	5285	0.06	5285	0.11	0	0.00
Thyroid cancer	822	0.01	330	0.02	492	0.01
Cervical cancer	1388	0.02	0	0.00	1388	0.04
Oral cavity cancer	3110	0.04	2541	0.05	569	0.02
Esophageal cancer	4666	0.06	4435	0.09	231	0.01
Laryngeal cancer	1019	0.01	952	0.02	67	0.002
**Cancer Incidence**						
All cancer	683614	8.18	412951	8.3	270663	8.01
Colorectal cancer	87769	1.05	59363	1.19	28406	0.84
Stomach cancer	111833	1.34	83624	1.68	28209	0.83
Lung cancer	66413	0.80	50389	1.01	16024	0.47
Breast cancer	47192	0.56	317	0.01	46875	1.39
Liver cancer	50168	0.60	39886	0.80	10282	0.30
Prostate cancer	39785	0.48	39785	0.80	0	0
Thyroid cancer	93869	1.12	26771	0.54	67098	1.99
Cervical cancer	8052	0.10	0	0	8052	0.24
Oral cavity cancer	11452	0.14	7850	0.16	3602	0.11
Esophageal cancer	7073	0.08	6594	0.13	479	0.01
Laryngeal cancer	4114	0.05	3927	0.08	187	0.01

Supplement table 4. Hazard ratios for the association of income and cancer incidence among men

Cancer incidence	Income	Model 1			Model 2			Model 3		
		HR	95% CI	p-trend	HR	95% CI	p-trend	HR	95% CI	p-trend
**All cancer**	Class 5 (low)	1.01	1.00-1.02	0.7197	0.99	0.98-1.00	<.0001	0.99	0.98-1.00	<.0001
	Class 4	0.98	0.97-0.99		0.96	0.95-0.97		0.96	0.95-0.97	
	Class 3	0.99	0.98-1.00		0.97	0.96-0.98		0.97	0.97-0.98	
	Class 2	0.99	0.98-1.00		0.98	0.97-0.99		0.98	0.97-0.99	
	Class 1 (high)	Ref.			Ref.			Ref.		
**Lung cancer**	Class 5 (low)	1.38	1.34-1.42	<.0001	1.21	1.18-1.25	<.0001	1.23	1.19-1.26	<.0001
	Class 4	1.30	1.26-1.34		1.14	1.11-1.17		1.13	1.10-1.17	
	Class 3	1.25	1.22-1.28		1.13	1.10-1.16		1.13	1.10-1.16	
	Class 2	1.13	1.10-1.16		1.06	1.03-1.09		1.06	1.03-1.09	
	Class 1 (high)	Ref.			Ref.			Ref.		
**Stomach cancer**	Class 5 (low)	1.04	1.01-1.06	0.0748	1.00	0.98-1.02	0.057	1.00	0.98-1.03	0.0714
	Class 4	0.99	0.96-1.01		0.96	0.94-0.98		0.96	0.93-0.98	
	Class 3	1.03	1.01-1.05		1.01	0.99-1.03		1.01	0.99-1.03	
	Class 2	1.02	1.00-1.04		1.01	0.99-1.03		1.00	0.99-1.02	
	Class 1 (high)	Ref.			Ref.			Ref.		
**Colorectal cancer**	Class 5 (low)	1.11	1.09-1.14	<.0001	1.10	1.07-1.13	<.0001	1.10	1.07-1.13	<.0001
	Class 4	1.00	0.97-1.02		0.99	0.97-1.02		1.00	0.97-1.02	
	Class 3	0.99	0.97-1.01		0.99	0.97-1.01		0.99	0.97-1.02	
	Class 2	0.98	0.95-1.00		0.97	0.95-1.00		0.98	0.96-1.00	
	Class 1 (high)	Ref.			Ref.			Ref.		
**Prostate cancer**	Class 5 (low)	0.70	0.67-0.72	<.0001	0.73	0.71-0.75	<.0001	0.73	0.70-0.75	<.0001
	Class 4	0.67	0.65-0.70		0.71	0.69-0.74		0.72	0.70-0.74	
	Class 3	0.71	0.69-0.73		0.75	0.72-0.77		0.75	0.73-0.77	
	Class 2	0.77	0.75-0.79		0.79	0.77-0.82		0.80	0.78-0.82	
	Class 1 (high)	Ref.			Ref.			Ref.		
**Liver cancer**	Class 5 (low)	1.17	1.13-1.21	<.0001	1.26	1.22-1.31	<.0001	1.28	1.24-1.33	<.0001
	Class 4	1.18	1.15-1.22		1.25	1.21-1.29		1.25	1.21-1.29	
	Class 3	1.16	1.13-1.19		1.20	1.17-1.24		1.20	1.17-1.23	
	Class 2	1.10	1.07-1.13		1.12	1.09-1.15		1.11	1.08-1.14	
	Class 1 (high)	Ref.			Ref.			Ref.		
	Class 5 (low)	0.34	0.32-0.36	<.0001	0.36	0.34-0.38	<.0001	0.35	0.33-0.37	<.0001
	Class 4	0.38	0.36-0.40		0.40	0.38-0.42		0.40	0.38-0.41	
**Thyroid cancer**	Class 3	0.54	0.53-0.56		0.56	0.54-0.58		0.55	0.54-0.57	
	Class 2	0.77	0.74-0.79		0.78	0.75-0.80		0.78	0.75-0.80	
	Class 1 (high)	Ref.			Ref.			Ref.		
	Class 5 (low)	1.09	1.01-1.18	0.0002	1.04	0.96-1.12	0.071	1.05	0.98-1.14	0.0416
	Class 4	1.16	1.08-1.25		1.09	1.02-1.17		1.08	1.01-1.17	
**Oral cancer**	Class 3	1.16	1.09-1.24		1.10	1.04-1.18		1.10	1.03-1.17	
	Class 2	1.11	1.04-1.18		1.08	1.01-1.15		1.07	1.00-1.14	
	Class 1 (high)	Ref.			Ref.			Ref.		
	Class 5 (low)	1.29	1.19-1.40	<.0001	1.15	1.06-1.25	<.0001	1.17	1.08-1.27	<.0001
	Class 4	1.36	1.26-1.47		1.17	1.09-1.26		1.17	1.08-1.26	
**Esophageal cancer**	Class 3	1.29	1.21-1.39		1.15	1.07-1.23		1.15	1.07-1.23	
	Class 2	1.12	1.04-1.20		1.04	0.97-1.12		1.04	0.97-1.12	
	Class 1 (high)	Ref.			Ref.			Ref.		
	Class 5 (low)	1.32	1.20-1.47	<.0001	1.16	1.05-1.28	0.002	1.17	1.05-1.29	0.0014
	Class 4	1.28	1.16-1.41		1.11	1.01-1.23		1.11	1.01-1.23	
**Laryngeal cancer**	Class 3	1.20	1.09-1.31		1.08	0.98-1.18		1.08	0.99-1.18	
	Class 2	1.12	1.02-1.22		1.04	0.95-1.14		1.05	0.96-1.15	
	Class 1 (high)	Ref.			Ref.			Ref.		

Model 1 adjusted for age.
Model 2 adjusted for age, CCI, smoking status, alcohol consumption, BMI, physical activity, and hepatitis virus for liver cancer.
Model 3 adjusted for all covariates, including variables in model 2, area, and insurance subscriber types.

Supplement table 5. Hazard ratios for the association of income and all-cause and cancer mortality among men

	Income	Model 1			Model 2			Model 3		
		HR	95% CI	p-trend	HR	95% CI	p-trend	HR	95% CI	p-trend
**All-cause mortality**	Class 5 (low)	1.66	1.65-1.68	<.0001	1.57	1.56-1.59	<.0001	1.61	1.60-1.63	<.0001
	Class 4	1.65	1.63-1.66		1.52	1.51-1.53		1.51	1.49-1.52	
	Class 3	1.47	1.46-1.48		1.37	1.36-1.39		1.37	1.36-1.38	
	Class 2	1.27	1.26-1.28		1.21	1.20-1.22		1.21	1.20-1.22	
	Class 1 (high)	Ref.			Ref.			Ref.		
**Cancer mortality**										
All cancer	Class 5 (low)	1.47	1.45-1.49	<.0001	1.38	1.35-1.40	<.0001	1.40	1.37-1.42	<.0001
	Class 4	1.41	1.39-1.44		1.31	1.29-1.33		1.30	1.28-1.32	
	Class 3	1.32	1.30-1.34		1.24	1.23-1.26		1.24	1.23-1.26	
	Class 2	1.19	1.17-1.21		1.14	1.13-1.16		1.14	1.13-1.16	
	Class 1 (high)	Ref.			Ref.			Ref.		
Lung cancer	Class 5 (low)	1.66	1.62-1.71	<.0001	1.44	1.40-1.48	<.0001	1.46	1.42-1.51	<.0001
	Class 4	1.57	1.52-1.61		1.33	1.30-1.37		1.32	1.29-1.36	
	Class 3	1.47	1.43-1.50		1.30	1.26-1.33		1.29	1.26-1.33	
	Class 2	1.28	1.25-1.32		1.18	1.15-1.22		1.18	1.15-1.21	
	Class 1 (high)	Ref.			Ref.			Ref.		
Stomach cancer	Class 5 (low)	1.64	1.57-1.71	<.0001	1.54	1.48-1.61	<.0001	1.56	1.49-1.63	<.0001
	Class 4	1.50	1.44-1.57		1.40	1.34-1.46		1.39	1.33-1.46	
	Class 3	1.41	1.36-1.47		1.34	1.29-1.39		1.33	1.28-1.39	
	Class 2	1.23	1.18-1.29		1.19	1.14-1.24		1.19	1.14-1.24	
	Class 1 (high)	Ref.			Ref.			Ref.		
Colorectal cancer	Class 5 (low)	1.54	1.46-1.62	<.0001	1.50	1.42-1.58	<.0001	1.5	1.43-1.58	<.0001
	Class 4	1.38	1.31-1.45		1.33	1.27-1.40		1.34	1.27-1.41	
	Class 3	1.27	1.22-1.34		1.24	1.19-1.30		1.25	1.19-1.31	
	Class 2	1.15	1.09-1.20		1.13	1.07-1.18		1.13	1.08-1.18	
	Class 1 (high)	Ref.			Ref.			Ref.		
Prostate cancer	Class 5 (low)	1.16	1.06-1.26	<.0001	1.14	1.04-1.24	0.0002	1.15	1.05-1.25	0.0002
	Class 4	1.17	1.07-1.27		1.14	1.05-1.24		1.13	1.04-1.23	
	Class 3	1.16	1.07-1.26		1.14	1.05-1.23		1.13	1.05-1.23	
	Class 2	1.08	1.00-1.17		1.06	0.98-1.15		1.06	0.98-1.15	
	Class 1 (high)	Ref.			Ref.			Ref.		
Liver cancer	Class 5 (low)	1.48	1.42-1.54	<.0001	1.54	1.48-1.60	<.0001	1.58	1.52-1.64	<.0001
	Class 4	1.47	1.42-1.53		1.50	1.44-1.55		1.50	1.44-1.55	
	Class 3	1.37	1.32-1.42		1.38	1.33-1.43		1.38	1.33-1.43	
	Class 2	1.23	1.19-1.27		1.23	1.19-1.27		1.23	1.19-1.27	
	Class 1 (high)	Ref.			Ref.			Ref.		
Thyroid cancer	Class 5 (low)	1.34	0.95-1.87	0.3522	1.29	0.92-1.81	0.4843	1.29	0.92-1.82	0.4859
	Class 4	1.10	0.77-1.56		1.07	0.75-1.52		1.07	0.75-1.53	
	Class 3	0.92	0.66-1.28		0.90	0.64-1.26		0.91	0.65-1.27	
	Class 2	1.36	1.02-1.83		1.34	1.00-1.81		1.36	1.01-1.82	
	Class 1 (high)	Ref.			Ref.			Ref.		
Oral cancer	Class 5 (low)	1.53	1.35-1.74	<.0001	1.38	1.22-1.57	<.0001	1.42	1.25-1.62	<.0001
	Class 4	1.54	1.36-1.74		1.36	1.20-1.54		1.36	1.20-1.54	
	Class 3	1.44	1.29-1.61		1.30	1.16-1.46		1.32	1.17-1.47	
	Class 2	1.27	1.13-1.43		1.20	1.06-1.34		1.20	1.07-1.35	
	Class 1 (high)	Ref.			Ref.			Ref.		
Esophageal cancer	Class 5 (low)	1.57	1.43-1.73	<.0001	1.38	1.25-1.52	<.0001	1.40	1.27-1.55	<.0001
	Class 4	1.79	1.64-1.96		1.50	1.37-1.64		1.49	1.36-1.63	
	Class 3	1.56	1.43-1.70		1.35	1.23-1.47		1.34	1.23-1.46	
	Class 2	1.32	1.21-1.45		1.21	1.10-1.32		1.20	1.10-1.32	
	Class 1 (high)	Ref.			Ref.			Ref.		
Laryngeal cancer	Class 5 (low)	2.21	1.81-2.69	<.0001	1.90	1.55-2.31	<.0001	1.91	1.57-2.34	<.0001
	Class 4	1.88	1.53-2.30		1.52	1.24-1.86		1.49	1.21-1.83	
	Class 3	1.79	1.48-2.17		1.51	1.25-1.83		1.50	1.23-1.81	
	Class 2	1.31	1.06-1.60		1.17	0.95-1.44		1.16	0.95-1.43	
	Class 1 (high)	Ref.			Ref.			Ref.		

Model 1 adjusted for age. Model 2 adjusted for age, CCI, smoking status, alcohol consumption, BMI, physical activity, and hepatitis virus for liver cancer. Model 3 adjusted for all covariates, including variables in model 2, area, and insurance subscriber types.

Supplement table 6. Hazard ratios for the association of income and cancer incidence among women

Cancer incidence	Income	Model 1			Model 2			Model 3		
		HR	95% CI	p-trend	HR	95% CI	p-trend	HR	95% CI	p-trend
**All cancer**	Class 5 (low)	0.86	0.85-0.87	<.0001	0.86	0.85-0.87	<.0001	0.86	0.85-0.87	<.0001
	Class 4	0.87	0.86-0.88		0.88	0.87-0.89		0.88	0.87-0.89	
	Class 3	0.92	0.91-0.93		0.93	0.92-0.94		0.93	0.92-0.94	
	Class 2	0.97	0.96-0.98		0.97	0.96-0.98		0.97	0.96-0.99	
	Class 1 (high)	Ref.			Ref.			Ref.		
**Breast cancer**	Class 5 (low)	0.69	0.67-0.71	<.0001	0.70	0.68-0.72	<.0001	0.70	0.68-0.72	<.0001
	Class 4	0.67	0.65-0.69		0.68	0.66-0.70		0.69	0.67-0.71	
	Class 3	0.76	0.74-0.78		0.77	0.75-0.80		0.78	0.76-0.80	
	Class 2	0.93	0.91-0.96		0.94	0.92-0.97		0.95	0.92-0.97	
	Class 1 (high)	Ref.			Ref.			Ref.		
**Thyroid cancer**	Class 5 (low)	0.65	0.63-0.67	<.0001	0.66	0.65-0.68	<.0001	0.66	0.65-0.68	<.0001
	Class 4	0.64	0.63-0.66		0.66	0.64-0.68		0.66	0.65-0.68	
	Class 3	0.75	0.73-0.77		0.76	0.75-0.78		0.76	0.75-0.78	
	Class 2	0.88	0.86-0.90		0.88	0.86-0.90		0.89	0.87-0.91	
	Class 1 (high)	Ref.			Ref.			Ref.		
**Colorectal cancer**	Class 5 (low)	0.95	0.92-0.99	0.006	0.95	0.91-0.98	0.003	0.95	0.92-0.99	0.007
	Class 4	0.97	0.94-1.01		0.97	0.94-1.01		0.98	0.94-1.01	
	Class 3	0.98	0.95-1.02		0.98	0.95-1.02		0.99	0.95-1.02	
	Class 2	0.98	0.95-1.02		0.98	0.95-1.02		0.99	0.95-1.02	
	Class 1 (high)	Ref.			Ref.			Ref.		
**Stomach cancer**	Class 5 (low)	0.97	0.94-1.01	0.12	0.97	0.93-1.00	0.048	0.96	0.93-1.00	0.043
	Class 4	1.00	0.97-1.04		1.00	0.96-1.03		1.00	0.96-1.03	
	Class 3	1.02	0.99-1.06		1.02	0.98-1.06		1.02	0.98-1.05	
	Class 2	1.02	0.98-1.05		1.02	0.98-1.05		1.01	0.98-1.05	
	Class 1 (high)	Ref.			Ref.			Ref.		
**Lung cancer**	Class 5 (low)	0.96	0.92-1.01	0.09	0.94	0.89-0.98	0.003	0.94	0.90-0.99	0.005
	Class 4	0.94	0.89-0.98		0.91	0.87-0.96		0.91	0.87-0.96	
	Class 3	0.99	0.95-1.04		0.98	0.94-1.03		0.98	0.94-1.03	
	Class 2	0.94	0.90-0.99		0.94	0.90-0.98		0.94	0.90-0.99	
	Class 1 (high)	Ref.			Ref.			Ref.		
**Liver cancer**	Class 5 (low)	1.16	1.09-1.23	<.0001	1.22	1.15-1.29	<.0001	1.23	1.16-1.30	<.0001
	Class 4	1.14	1.08-1.22		1.18	1.11-1.26		1.18	1.11-1.25	
	Class 3	1.14	1.07-1.20		1.17	1.10-1.24		1.16	1.10-1.24	
	Class 2	1.08	1.02-1.14		1.10	1.04-1.17		1.10	1.03-1.17	
	Class 1 (high)	Ref.			Ref.			Ref.		
**Cervical cancer**	Class 5 (low)	1.46	1.36-1.56	<.0001	1.43	1.33-1.53	<.0001	1.47	1.37-1.57	<.0001
	Class 4	1.36	1.26-1.45		1.33	1.24-1.43		1.35	1.26-1.45	
	Class 3	1.16	1.08-1.24		1.14	1.07-1.23		1.16	1.08-1.25	
	Class 2	1.10	1.02-1.18		1.09	1.02-1.18		1.10	1.02-1.18	
	Class 1 (high)	Ref.			Ref.			Ref.		

Model 1 adjusted for age.
Model 2 adjusted for age, CCI, smoking status, alcohol consumption, BMI, physical activity, and hepatitis virus for liver cancer.
Model 3 adjusted for all covariates, including variables in model 2, area, and insurance subscriber types.

Supplement table 7. Hazard ratios for the association of income and all-cause mortality and cancer mortality among women

	Income	Model 1			Model 2			Model 3		
		HR	95% CI	p-trend	HR	95% CI	p-trend	HR	95% CI	p-trend
**All-cause mortality**	Class 5 (low)	1.27	1.25-1.28	<.0001	1.24	1.22-1.25	<.0001	1.21	1.20-1.22	<.0001
	Class 4	1.25	1.23-1.26		1.21	1.20-1.23		1.18	1.17-1.20	
	Class 3	1.17	1.16-1.19		1.15	1.13-1.16		1.13	1.12-1.14	
	Class 2	1.10	1.09-1.11		1.08	1.07-1.10		1.08	1.06-1.09	
	Class 1 (high)	Ref.			Ref.			Ref.		
**Cancer mortality**										
**All cancer death**	Class 5 (low)	1.15	1.12-1.17	<.0001	1.13	1.10-1.15	<.0001	1.12	1.10-1.15	<.0001
	Class 4	1.15	1.12-1.17		1.12	1.10-1.15		1.12	1.09-1.14	
	Class 3	1.09	1.06-1.11		1.07	1.05-1.10		1.07	1.05-1.10	
	Class 2	1.05	1.03-1.08		1.04	1.02-1.07		1.04	1.02-1.07	
	Class 1 (high)	Ref.			Ref.			Ref.		
**Breast cancer mortality**	Class 5 (low)	1.01	0.91-1.12	0.453	1.01	0.91-1.12	0.484	1.02	0.92-1.13	0.411
	Class 4	1.04	0.94-1.15		1.04	0.94-1.15		1.05	0.94-1.16	
	Class 3	1.01	0.92-1.12		1.02	0.92-1.12		1.02	0.92-1.13	
	Class 2	0.97	0.87-1.07		0.97	0.87-1.07		0.97	0.88-1.08	
	Class 1 (high)	Ref.			Ref.			Ref.		
**Thyroid cancer mortality**	Class 5 (low)	1.41	1.09-1.84	0.03	1.44	1.11-1.87	0.02	1.48	1.14-1.93	0.011
	Class 4	1.20	0.90-1.60		1.22	0.92-1.63		1.27	0.95-1.70	
	Class 3	1.19	0.90-1.56		1.20	0.91-1.58		1.23	0.93-1.62	
	Class 2	1.30	0.99-1.70		1.31	1.00-1.71		1.33	1.02-1.74	
	Class 1 (high)	Ref.			Ref.			Ref.		
**Colorectal cancer mortality**	Class 5 (low)	1.14	1.07-1.22	<.0001	1.13	1.05-1.20	0.0002	1.12	1.05-1.20	0.0003
	Class 4	1.15	1.07-1.23		1.13	1.05-1.21		1.12	1.05-1.20	
	Class 3	1.07	1.00-1.14		1.06	0.99-1.13		1.05	0.98-1.12	
	Class 2	1.09	1.02-1.17		1.08	1.01-1.16		1.08	1.01-1.15	
	Class 1 (high)	Ref.			Ref.			Ref.		
**Stomach cancer mortality**	Class 5 (low)	1.22	1.14-1.30	<.0001	1.18	1.11-1.27	<.0001	1.18	1.10-1.26	<.0001
	Class 4	1.22	1.14-1.31		1.19	1.11-1.27		1.18	1.10-1.26	
	Class 3	1.08	1.01-1.16		1.06	0.99-1.13		1.05	0.98-1.12	
	Class 2	1.11	1.04-1.19		1.10	1.03-1.18		1.09	1.02-1.17	
	Class 1 (high)	Ref.			Ref.			Ref.		
**Lung cancer mortality**	Class 5 (low)	1.22	1.16-1.29	<.0001	1.15	1.09-1.22	<.0001	1.15	1.09-1.21	<.0001
	Class 4	1.17	1.11-1.24		1.11	1.05-1.18		1.1	1.04-1.16	
	Class 3	1.15	1.09-1.21		1.11	1.05-1.17		1.1	1.05-1.17	
	Class 2	1.00	0.95-1.06		0.98	0.93-1.04		0.98	0.93-1.04	
	Class 1 (high)	Ref.			Ref.			Ref.		
**Liver cancer mortality**	Class 5 (low)	1.30	1.21-1.39	<.0001	1.33	1.24-1.43	<.0001	1.33	1.24-1.42	<.0001
	Class 4	1.32	1.23-1.42		1.34	1.25-1.44		1.32	1.22-1.42	
	Class 3	1.22	1.14-1.31		1.24	1.15-1.33		1.23	1.14-1.32	
	Class 2	1.18	1.10-1.26		1.19	1.11-1.27		1.18	1.10-1.27	
	Class 1 (high)	Ref.			Ref.			Ref.		
**Cervical cancer mortality**	Class 5 (low)	1.58	1.36-1.84	<.0001	1.51	1.30-1.77	<.0001	1.53	1.31-1.79	<.0001
	Class 4	1.29	1.09-1.52		1.23	1.04-1.46		1.23	1.04-1.46	
	Class 3	1.19	1.01-1.40		1.15	0.98-1.36		1.16	0.98-1.37	
	Class 2	0.96	0.81-1.15		0.95	0.79-1.13		0.95	0.80-1.14	
	Class 1 (high)	Ref.			Ref.			Ref.		

Model 1 adjusted for age.
Model 2 adjusted for age, CCI, smoking status, alcohol consumption, BMI, physical activity, and hepatitis virus for liver cancer.
Model 3 adjusted for all covariates, including variables in model 2, area, and insurance subscriber types.

Supplement table 8. Odd ratios of unhealthy lifestyle behavior among income classes for both sexes

Income	Class 1 (highest)	Class 2	Class 3	Class 4	Class 5 (lowest)	p-trend
**Unhealthy lifestyle**						
Ever smoking	*Ref.*	1.10<br>1.09-1.10	1.17<br>1.16-1.17	1.23<br>1.22-1.24	1.23<br>1.22-1.24	<.0001
Heavy drinking	*Ref.*	1.12<br>1.11-1.13	1.23<br>1.22-1.24	1.31<br>1.30-1.32	1.33<br>1.32-1.34	<.0001
Physically inactive	*Ref.*	1.34<br>1.33-1.35	1.63<br>1.63-1.64	1.80<br>1.79-1.81	1.78<br>1.77-1.79	<.0001
Unhealthy BMI	*Ref.*	1.01<br>1.00-1.01	0.97<br>0.97-0.97	0.91<br>0.91-0.92	0.96<br>0.95-0.96	<.0001
Extreme BMI (BMI<18.5 or ≥30)	*Ref.*	1.13<br>1.12-1.15	1.32<br>1.31-1.33	1.46<br>1.44-1.47	1.45<br>1.44-1.47	<.0001
**Unhealthy score**						
≥ 1	*Ref.*	1.29<br>1.28-1.30	1.52<br>1.51-1.53	1.66<br>1.65-1.67	1.67<br>1.66-1.68	<.0001
≥ 2	*Ref.*	1.20<br>1.19-1.21	1.33<br>1.32-1.33	1.37<br>1.36-1.37	1.37<br>1.37-1.38	<.0001
≥ 3	*Ref.*	1.22<br>1.21-1.22	1.35<br>1.34-1.36	1.38<br>1.37-1.39	1.42<br>1.41-1.43	<.0001
≥ 4	*Ref.*	1.29<br>1.26-1.31	1.52<br>1.49-1.55	1.55<br>1.52-1.58	1.62<br>1.58-1.66	<.0001

Model was adjusted for age, sex, area, insurance subscriber types, and CCI.

Supplement figure 1. Joint association of income and combined unhealthy lifestyle on cancer incidence among men[a]

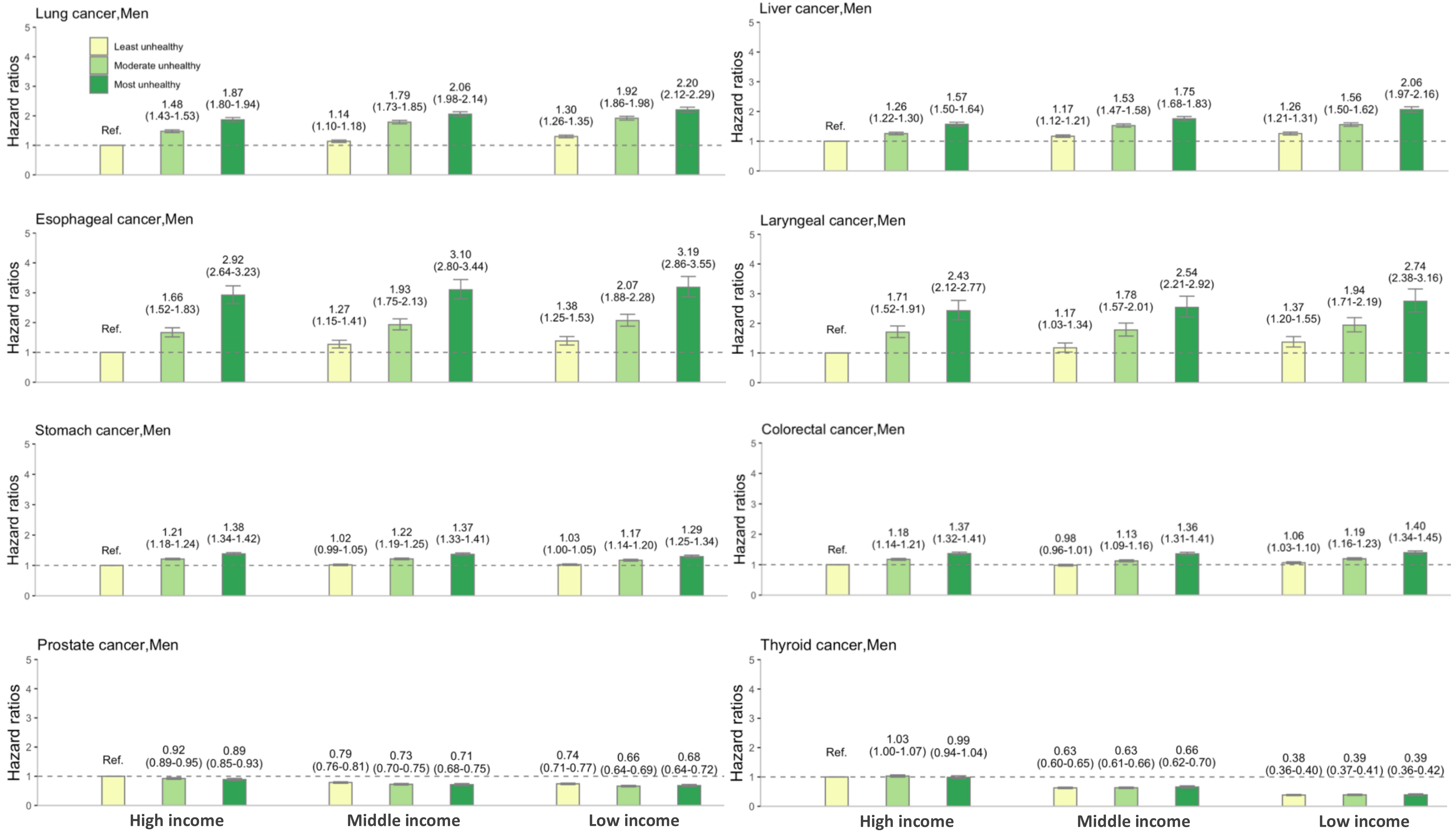

[a]*Hazard ratios adjusted for age, CCI, area and insurance subscriber types, and chronic viral hepatitis for liver cancer when appropriate.*

Supplement figure 2. Joint association of income and combined unhealthy lifestyle on cancer incidence among women[a]

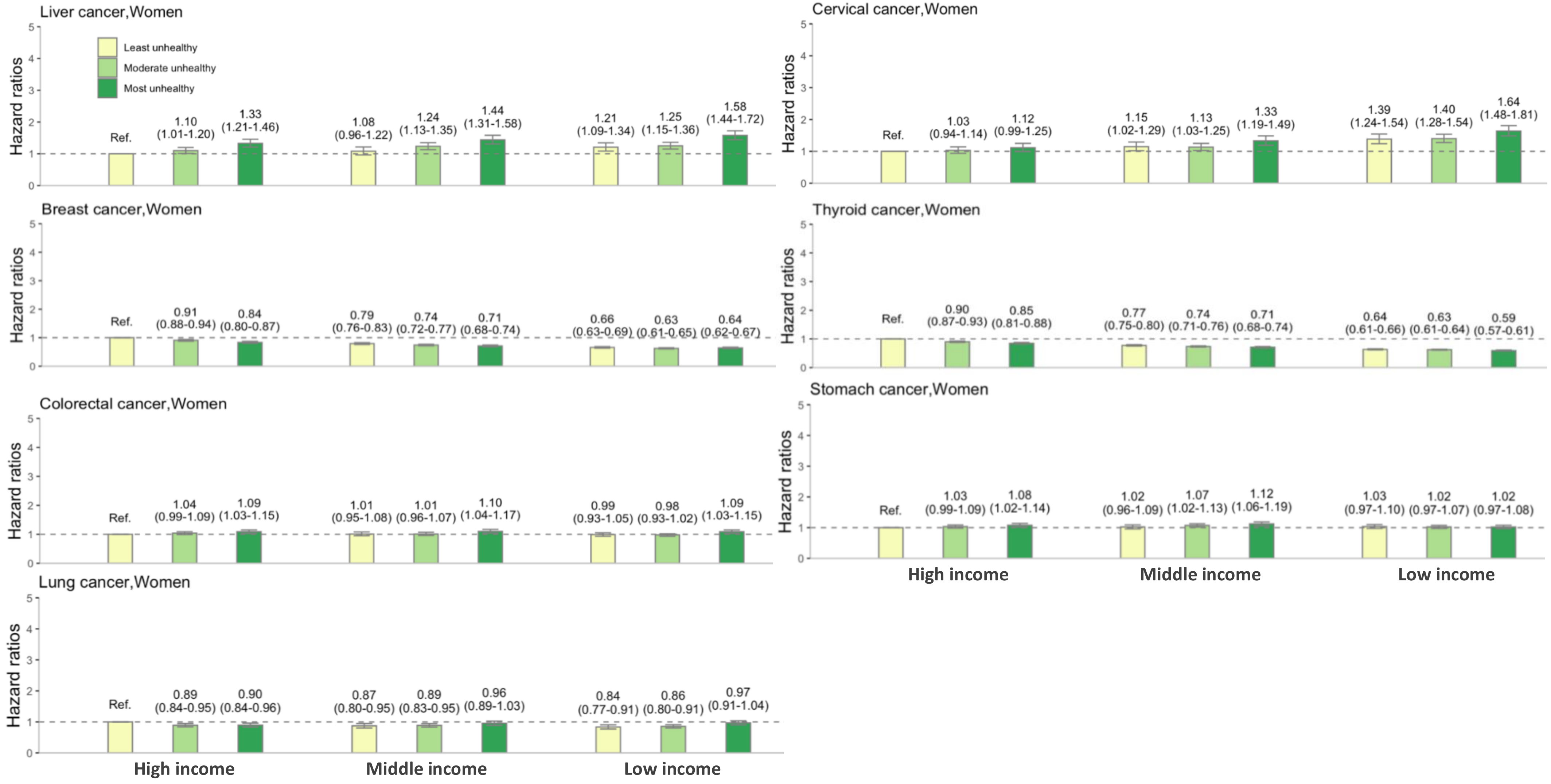

[a]*Hazard ratios adjusted for age, CCI, area and insurance subscriber types, and chronic viral hepatitis for liver cancer when appropriate.*

Supplement Figure 3. Joint effect of income and unhealthy lifestyle on all-cause mortality among men and women

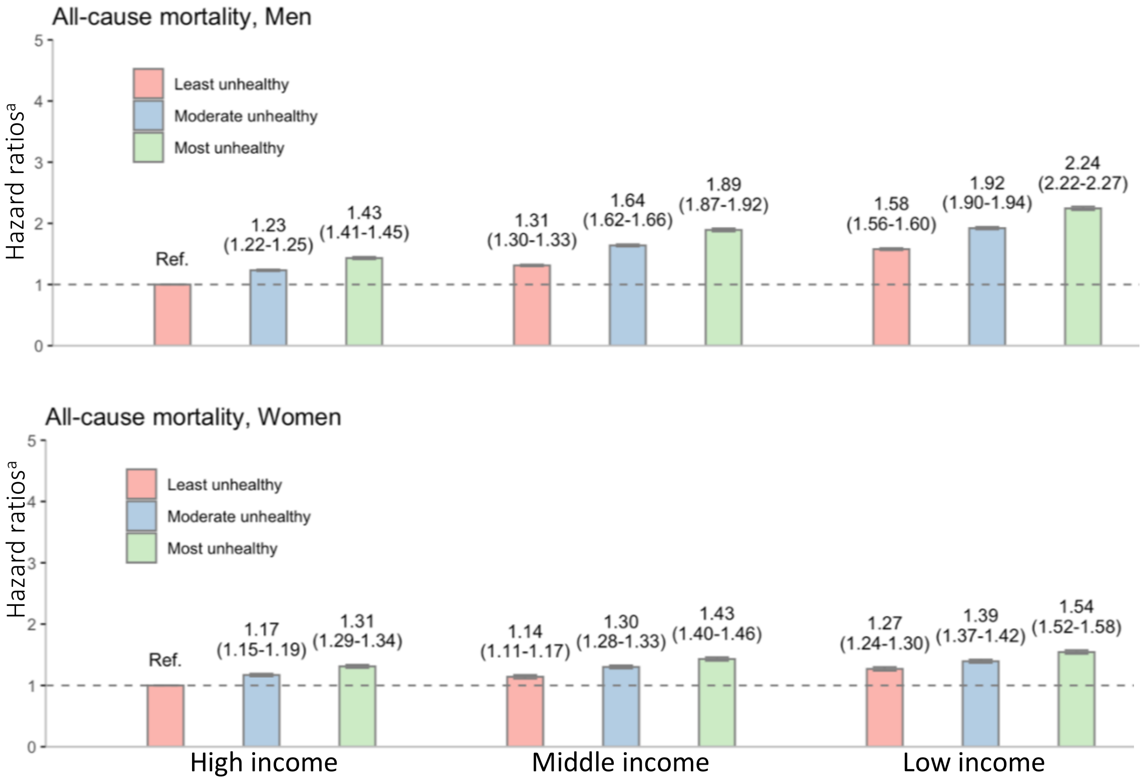

[a]*Hazard ratios adjusted for age, residential area, insurance subscriber types, and CCI.*
